# Plant-Derived Nanocarriers for Drug Delivery: A Unified Framework Integrating Extracellular Vesicles, Engineered Phytocarriers, Hybrid Platforms, and Bioinspired Systems

**DOI:** 10.3390/plants15060908

**Published:** 2026-03-15

**Authors:** Adina-Elena Segneanu, George Dan Mogoşanu, Cornelia Bejenaru, Roxana Kostici, Ludovic Everard Bejenaru

**Affiliations:** 1 Department of Chemistry, Faculty of Chemistry, Biology, and Geography, West University of Timişoara, 16 Johann Heinrich Pestalozzi Street, 300115 Timişoara, Romania; adina.segneanu@e-uvt.ro; 2 Department of Chemistry, Institute for Advanced Environmental Research, West University of Timişoara (ICAM–WUT), 4 Oituz Street, 300086 Timişoara, Romania; 3 Drug Research Center, Faculty of Pharmacy, University of Medicine and Pharmacy of Craiova, 2 Petru Rareş Street, 200349 Craiova, Romania; george.mogosanu@umfcv.ro (G.D.M.); ludovic.bejenaru@umfcv.ro (L.E.B.); 4 Department of Pharmacognosy & Phytotherapy, Faculty of Pharmacy, University of Medicine and Pharmacy of Craiova, 2 Petru Rareş Street, 200349 Craiova, Romania; 5 Department of Pharmaceutical Botany, Faculty of Pharmacy, University of Medicine and Pharmacy of Craiova, 2 Petru Rareş Street, 200349 Craiova, Romania; 6 Department of Toxicology, Faculty of Pharmacy, University of Medicine and Pharmacy of Craiova, 2 Petru Rareş Street, 200349 Craiova, Romania

**Keywords:** plant, nanocarriers, drug delivery, extracellular vesicles, phytocarriers, hybrid platforms, bioinspired systems

## Abstract

Plant-derived extracellular vesicles (PDEVs), engineered phytosomes, bioinspired polymeric plant-based nanoparticles (PBNPs), hybrid phyto-inorganic nanocomposites, green-synthesized metal nanoparticles, self-assembled nanoarchitectures, and multifunctional composites represent a rapidly advancing class of sustainable, nature-inspired nanocarriers. These platforms combine exceptional biocompatibility, negligible immunogenicity, and renewable sourcing with tunable drug loading, targeted delivery, and controlled release properties. This review synthesizes translational advances from 2020 to 2026, covering scalable isolation/bioprocessing (bioreactors, elicitation), multi-parametric physicochemical/multi-omics characterization, rational engineering/hybridization, and rigorous in vitro/in vivo assessments of uptake, biodistribution, pharmacokinetic (PK), and efficacy. Phytosomes and PBNPs markedly enhance oral bioavailability and targeted delivery of lipophilic phytochemicals, while PDEVs offer unique immunomodulatory, anti-inflammatory, and gene-regulatory activities. Hybrid and green-synthesized systems provide structural stability, redox modulation, and synergistic effects, and self-assembled/multifunctional composites address solubilization barriers with stimuli-responsive design. Early-phase human studies on grapefruit-, ginger-, turmeric-, and ginseng-derived PDEVs report excellent short-term safety, favorable PK, and preliminary bioactivity signals, with no observed immunogenicity or dose-limiting toxicities; however, these trials remain exploratory, constrained by small sample sizes and safety-focused endpoints. Despite challenges, including methodological heterogeneity, variable yields, long-term safety uncertainties (notably for inorganic hybrids), and regulatory ambiguities, emerging strategies such as clustered regularly interspaced short palindromic repeats (CRISPR)-engineered plant line; artificial-intelligence-driven process optimization; standardized guidelines, and integrated clinical, intellectual property, and commercialization frameworks are progressively addressing these barriers. Collectively, these advances position plant-derived nanocarriers as immunologically privileged, eco-friendly alternatives to synthetic and mammalian platforms, laying the foundation for a sustainable era of precision phytomedicine.

## 1. Introduction

The integration of plant-based nanotechnology into modern drug delivery reflects the convergence of traditional pharmacognosy and nanoscale formulation science. Medicinal plant use has been documented across ancient Egyptian, Chinese, and Greco-Roman systems [1,2], and contemporary advances in nanotechnology have enabled renewed investigation of plant-derived materials as functional delivery platforms [3,4].

Plant-derived nanocarriers are generally characterized by biocompatibility, low observed immunogenicity in preclinical systems, renewable sourcing, and the presence of intrinsically bioactive phytochemical cargo. These properties have prompted evaluation of their potential to address long-standing formulation challenges associated with both phytochemicals and synthetic agents, including limited aqueous solubility, restricted membrane permeability, chemical instability, rapid metabolic clearance, and suboptimal target localization [3,4,5,6].

This review systematically examines three complementary and progressive translational classes of plant-derived carrier systems: plant-derived extracellular vesicles (PDEVs), engineered phyto-nanocarriers, and bioinspired polymeric plant-based nanoparticles (PBNPs).

### 1.1. Plant-Derived Extracellular Vesicles

PDEVs, also referred to as plant-derived exosome-like nanoparticles (PELNPs), are lipid bilayer nanostructures typically ranging from 30 to 500 nm in diameter. They are released into apoplastic fluids, vascular sap, root exudates, or extracellular matrices and contain heterogeneous molecular cargo, including proteins, phospholipids (e.g., phosphatidylcholine (PC), phosphatidylethanolamine (PE), glycosyl-inositol-phospho-ceramides (GIPCs)), small ribonucleic acids (RNAs) (e.g., miR-156, miR-159), and plant secondary metabolites [7,8,9,10,11].

Initially characterized for roles in plant intercellular communication and stress responses, PDEVs have since been investigated for their capacity to interact with mammalian cells following oral or parenteral administration [12,13,14]. Experimental studies report gastrointestinal (GI) stability, limited acute immunogenicity, and the ability to transport bioactive molecules across biological barriers. These properties support their evaluation both as biologically active entities and as nanoscale delivery vehicles in preclinical models of inflammatory disorders, oncology, central nervous system (CNS) diseases, dermatological conditions, and metabolic dysfunction [9,12,15,16,17,18,19,20].

Recent comparative investigations have further highlighted the distinctive advantages of PDEVs relative to mammalian vesicle systems in the context of therapeutic delivery. In addition to their renewable botanical origin and scalable production, PDEVs exhibit favorable safety characteristics, including minimal risk of pathogen transmission, low intrinsic immunogenicity, and compatibility with oral administration routes. Importantly, their molecular cargo (comprising plant-specific lipids, proteins, metabolites, and small RNAs) enables cross-kingdom biological communication and functional modulation of mammalian cellular pathways. These properties collectively position PDEVs as biologically active nanocarriers capable of simultaneously acting as therapeutic entities and delivery platforms. Comparative analyses of plant vs. mammalian extracellular vesicles (EVs) further emphasize the translational potential of PDEVs as sustainable and immunologically privileged alternatives for drug delivery and nanomedicine applications [21].

Advances in isolation and characterization methodologies, including differential ultracentrifugation (DUC), size-exclusion chromatography (SEC), tangential flow filtration (TFF), and controlled cultivation systems, have improved recovery and analytical consistency [22,23,24,25,26,27,28,29,30,31]. Parallel implementation of proteomics, lipidomics, small RNA sequencing, and metabolomics has refined molecular profiling and functional interpretation, although standardization across laboratories remains incomplete [32].

### 1.2. Engineered Phyto-Nanocarriers

Engineered phyto-nanocarriers represent structurally defined formulations developed to improve solubility, membrane interaction, systemic exposure, and pharmacokinetic (PK) behavior of plant-derived bioactive compounds [5,33,34,35]. Unlike traditional extracts, these systems aim to control physicochemical properties and release kinetics while preserving phytochemical integrity.

#### 1.2.1. Phytosomes and Phospholipid Complexes

Phytosomes are non-covalent molecular complexes formed between phospholipids, most commonly PC, and polyphenolic or terpenoid compounds. Complexation enhances membrane affinity and improves oral absorption relative to unformulated phytochemicals [36,37,38,39,40,41,42,43,44,45].

Unlike conventional liposomes, in which compounds are passively entrapped, phytosomes involve defined lipid–molecule associations that may improve partitioning and reduce enzymatic degradation [37,39,42].

Clinically investigated examples include curcumin (Meriva^®^) and silybin (Siliphos^®^), which demonstrate increased plasma exposure compared with free compounds in human PK studies [37,42]. These data support lipid–polyphenol complexation as a reproducible strategy for bioavailability enhancement.

#### 1.2.2. Lipid Nanoparticles and Hybrid Lipid Platforms

Solid lipid nanoparticles (SLNPs) and nanostructured lipid carriers (NLCs) incorporate phytochemicals within semi-crystalline or mixed lipid matrices. These systems can provide sustained release, improved solubilization of hydrophobic compounds, and protection against oxidative degradation [46].

Preclinical models report improved systemic exposure and enhanced pharmacodynamic (PD) responses compared with free phytochemicals. NLCs, which incorporate liquid lipids within the matrix, generally permit higher loading capacity than SLNPs and reduce expulsion during storage [5,35,46].

#### 1.2.3. Green Synthesized Metal and Metal Oxide Nanoparticles

Green synthesis approaches use plant extracts as reducing and stabilizing agents to generate metal/metal oxide nanoparticles (NPs), e.g., mesoporous silica, silver (Ag), gold (Au), magnetite (Fe_3_O_4_), and zinc oxide (ZnO) [47,48,49,50,51,52,53]. Phytochemicals adsorbed onto the NP surface may contribute to biological activity while influencing colloidal stability and ion release kinetics.

These systems have been explored primarily in antimicrobial, cytotoxic, and anti-inflammatory models [48,49,54,55,56,57]. Although synergistic effects have been reported in vitro, long-term safety, biodistribution, and regulatory classification of inorganic components remain active areas of investigation.

#### 1.2.4. Hybrid Phyto-Inorganic Nanocomposites

Hybrid composites integrate plant-derived molecules with inorganic matrices (e.g., mesoporous silica, zeolites, Fe_3_O_4_) or polymeric scaffolds to modulate release profiles and stability [58,59,60,61].

Examples include phenolic-loaded clinoptilolite systems and turmeric-derived composites assembled with metallic NPs [58,59,60,61]. These materials combine structural rigidity with phytochemical functionality, enabling controlled diffusion and surface-mediated interactions.

However, the mechanistic understanding of matrix–cargo interactions and in vivo behavior remains limited, and scalability requires further validation [36].

#### 1.2.5. Nanoemulsions and Multifunctional Dispersions

Phyto-nanoemulsions reduce droplet size to the nanoscale, improving dispersion stability and GI absorption of hydrophobic constituents [18,19,62,63]. High-pressure homogenization and low-energy emulsification methods support scalable production.

Hybridization with polymeric or inorganic components may further modify release kinetics and physical stability. Comparative benchmarking against lipid NPs and phytosomes remains limited.

#### 1.2.6. Self-Assembled Nanoarchitectures

Self-assembled systems, including micelles, supramolecular phytochemical aggregates, and nanoemulsified networks, leverage amphiphilic interactions, hydrogen bonding, and π–π stacking to enhance apparent solubility and bioavailability [35,38,39,40,64].

While these systems offer formulation flexibility and potential stimuli-responsive behavior, standardized comparative evaluation relative to lipid or polymer-based carriers is still emerging [34,35].

### 1.3. Bioinspired Plant-Based Polymeric Nanoparticles

Bioinspired PBNPs are formulated from plant proteins and polysaccharides to generate biodegradable and mucoadhesive delivery matrices [65,66].

Zein-based NPs, derived from maize prolamin, self-assemble into structures capable of encapsulating hydrophobic molecules and supporting sustained release [67,68,69,70]. Chitosan and plant polysaccharides provide cationic surfaces and pH-responsive behavior suitable for mucosal or colon-targeted delivery [66,71,72,73].

Composite formulations integrating lipidic or inorganic elements have been investigated to enhance mechanical stability and multifunctionality. However, long-term safety and inter-batch reproducibility require systematic evaluation relative to established synthetic polymers [36,74,75].

### 1.4. Knowledge Gaps and Conceptual Integration

Despite substantial diversification across PDEVs, lipid nanocarriers, polymeric matrices, green-synthesized NPs, and hybrid composites, the field remains methodologically heterogeneous.

Key limitations include (*i*) restricted cross-platform benchmarking under standardized physicochemical and biological conditions; (*ii*) incomplete mechanistic characterization of matrix–cargo interactions, particularly in hybrid and green synthesized systems [36,50,51,52,53,58,59,60,61]; (*iii*) variability in isolation and characterization protocols, especially for PDEVs [26,27,28,29,30,31,32,74]; (*iv*) sparse integration of multi-omics profiling with PK and functional datasets [33]; and (*v*) limited translational benchmarking addressing scalability, controlled release, long-term safety, and regulatory classification [5,36,42].

This review addresses these issues by consolidating naturally secreted vesicles, engineered lipid systems, polymeric matrices, and hybrid constructs within a unified analytical framework. By aligning structural features with reported functional outcomes, the approach facilitates systematic comparison without presuming equivalence across carrier classes.

## 2. Advantages of Plant-Based Nanocarrier Systems

Plant-derived nanocarriers encompass PDEVs, engineered lipid and polymer systems, and hybrid inorganic–organic constructs. Collectively, they offer renewable sourcing, compositional diversity, and formulation adaptability [3,4,5,6,7,8,19,35,37,38,39,46,47,61,74].

Preclinical studies frequently report acceptable short-term tolerability and improved systemic exposure of encapsulated phytochemicals relative to free compounds [7,8,9,15,76]. However, human efficacy data remain limited primarily to selected phytosome formulations.

Plant matrices may contribute to endogenous bioactive components (e.g., polyphenols, terpenoids, small RNAs) that complement carrier function. Whether these intrinsic components meaningfully contribute to therapeutic outcomes in humans remains to be established.

Large-scale reproducibility, Good Manufacturing Practice (GMP) standardization, and regulatory alignment remain essential steps for clinical translation.

### 2.1. Biosafety and Oral Compatibility

Many plant-based nanocarriers are derived from edible or traditionally consumed botanical sources, including *Citrus* × *paradisi*, *Zingiber officinale*, *Curcuma longa*, *Panax ginseng*, *Camellia sinensis*, *Moringa oleifera*, and *Trigonella foenum-graecum* [3,4,5,8,16]. Their established dietary exposure provides a favorable starting point for safety evaluation, although formulation-specific assessment remains essential.

In preclinical models, several systems, including PDEVs, phytosomes, SLNPs, and NLCs, have demonstrated structural stability under simulated GI conditions and acceptable tolerability during short-term exposure [7,8,15,76]. These findings support continued investigation of oral delivery strategies; however, robust clinical confirmation is limited.

### 2.2. Renewable Sourcing and Manufacturing Considerations

Plant biomass represents a renewable raw material source. Compared with mammalian cell–derived vesicles or complex synthetic nanomaterials, plant-based systems may reduce reliance on specialized culture infrastructure and hazardous reagents [48,58,59,76,77].

Nevertheless, scalability, inter-batch reproducibility, and compliance with GMP standards remain active areas of development. Standardization of extraction, purification, and characterization protocols is particularly important for vesicle-based and hybrid systems.

### 2.3. Immunological and Cytotoxicity Profiles

In vitro and animal studies generally report low induction of proinflammatory cytokines, e.g., interleukin-6 (IL-6), tumor necrosis factor-alpha (TNF-α), and minimal complement activation following administration of selected plant-derived vesicles or lipid-based phytocarriers [8,9]. Cytotoxicity assays frequently demonstrate high cell viability within tested concentration ranges under controlled experimental conditions [9,71,72,73,78].

While these data indicate acceptable short-term tolerability in preclinical settings, a comprehensive human safety evaluation, including immunogenicity, long-term biodistribution, and chronic exposure assessment, remains necessary.

Despite encouraging short-term preclinical safety data for green-synthesized metal and metal oxide NPs, such as low proinflammatory cytokine induction (e.g., IL-6, TNF-α), limited complement activation, and high cell viability in standard cytotoxicity assays for selected systems (e.g., plant-capped AgNPs, AuNPs, ZnO NPs, Fe_3_O_4_ NPs), significant gaps remain in the current understanding of their long-term safety and immunological profile. Most published evaluations emphasize acute or subacute tolerability in rodent models, with comparatively few studies addressing chronic exposure, long-term metabolism, biodistribution dynamics, and adaptive immune responses [79,80,81,82,83].

The broader green nanotechnology literature on plant-derived metal NPs underscores several underlying uncertainties. Green-synthesized NPs often exhibit reduced acute cytotoxicity relative to chemically synthesized counterparts, attributed to phytocapping agents that mitigate surface reactivity and metal ion release, but this does not obviate the need for extended in vivo characterization [84,85,86,87]. Specifically, chronic toxicity, organ accumulation and clearance kinetics, and longitudinal immunogenicity have not been systematically evaluated for many green-synthesized metal/metal oxide NPs:

- Chronic toxicity and organ accumulation: sustained exposure may lead to oxidative stress due to prolonged reactive oxygen species (ROS) generation, genotoxicity from slow metal ion leaching, or accumulation in reticuloendothelial organs such as the liver, spleen, and kidney [80,82,83]. These phenomena may not be captured in short-term studies but can influence long-term safety and functional integrity of critical tissues.
- In vivo metabolism and clearance: hepatic and renal clearance pathways for phytocapped metal NPs are poorly characterized, and species differences in metalloprotein binding and excretion further complicate safety extrapolation across models [79,81].
- Immunogenicity and adaptive responses: while early studies report minimal complement activation in vitro or after single doses, there is a lack of robust assessment of adaptive immune activation, antiparticle antibody (Ab) formation, or delayed hypersensitivity with repeated dosing [85,86].
- Variation in plant capping agents: phytochemical coronas formed during green synthesis can vary substantially depending on plant species and extraction conditions. These differences influence corona composition, protein adsorption, opsonization, biodistribution, and immune recognition, which can unpredictably affect long-term safety and clearance [82,83].

These limitations are not unique to a specific class of green-synthesized NPs but reflect widespread knowledge gaps identified in systematic reviews of green nanomaterials [80,81]. Until robust, Good Laboratory Practice (GLP)-compliant chronic toxicology studies, longitudinal biodistribution tracking (e.g., radiolabeling or inductively coupled plasma mass spectrometry), and comprehensive immunogenicity profiling, including T- and B-cell responses, complement pathways, and cytokine networks, are available, caution is warranted in translational applications of green-synthesized metal/metal oxide NPs and their phyto-inorganic hybrids. This cautionary stance is especially important given the manuscript’s positioning of these systems as “sustainable” and “biocompatible” alternatives to synthetic counterparts [3,4,88].

Although green-synthesized metal and metal oxide NPs show promising acute safety, Table 1 highlights that their long-term toxicology, biodistribution, and immunogenicity remain largely uncharacterized, underscoring the urgent need for standardized preclinical evaluation.

### 2.4. Intrinsic Phytochemical Contributions

Unlike fully synthetic carriers, plant-derived systems may retain endogenous phytochemicals (e.g., polyphenols, terpenoids, small RNAs) that could contribute to biological activity in addition to encapsulation functions.

Illustrative examples include (*i*) vesicles derived from *C. sinensis* associated with activation of antioxidant pathways in cellular models [89], (*ii*) silymarin phytosomes derived from *Silybum marianum* demonstrating modulation of fibrotic signaling pathways in experimental systems [90], (*iii*) curcumin-loaded lipid carriers and phytosomes based on *C. longa* showing enhanced anti-inflammatory responses compared with unformulated curcumin in preclinical models [8,12], (*iv*) mineral–plant hybrids (e.g., clinoptilolite–flavonoid composites) investigated for combined adsorption and antioxidant properties [13,58], and (*v*) Fe_3_O_4_-based plant-mediated NPs explored for magnetically guided delivery and antioxidant activity [59].

These observations suggest that plant matrices may provide functional components beyond passive transport. The relative contribution of intrinsic phytochemicals vs. carrier-mediated PK enhancement, however, requires systematic evaluation.

#### Mechanistic Delineation of Intrinsic Bioactivity vs. Carrier Function in PDEVs

PDEVs are increasingly recognized not only as biocompatible nanocarrier platforms but also as biologically active entities due to their endogenous cargo. Distinguishing between these two facets, intrinsic therapeutic activity and carrier-mediated delivery enhancement, is essential for accurate interpretation of experimental outcomes and for the rational design of PDEV-based nanotherapeutics.

Unlike synthetic delivery systems that are generally inert carriers until loaded with cargo, PDEVs inherently encapsulate a complex repertoire of biomolecules, including small regulatory RNAs, lipids, proteins, and plant secondary metabolites, that can influence biological pathways in recipient mammalian cells. These molecules can exert direct functional effects (e.g., gene expression modulation, signaling pathway activation, antioxidant activity) independent of any externally loaded therapeutic payload [5,9,10,11,12,91].

At the same time, the vesicular lipid bilayer architecture provides a delivery system that protects cargo from degradation, enhances cellular internalization, and contributes to tissue tropism and biodistribution characteristics [8,16,92,93,94,95].

Extensive studies increasingly recognize that the biological activity of PDEVs arises from two complementary mechanisms: (*i*) intrinsic bioactive cargo and (*ii*) carrier functions arising from vesicle structure.

Intrinsic bioactive cargo include:

- Small RNAs, e.g., plant microRNAs (miRNAs): plant miRNAs within PDEVs have been implicated in cross-kingdom regulatory interactions, modulating gene expression related to inflammatory signaling and metabolic processes following internalization by mammalian cells [9,11,12,13,14].
- Bioactive lipids: unique plant lipid profiles, such as high levels of phosphatidic acid (PA), PC, and PE, may influence membrane fusion events and preferential uptake by specific cell types, e.g., macrophages and epithelial cells, thereby modulating immune responses [10,11,95].
- Secondary metabolites: endogenous phytochemicals such as gingerols from ginger vesicles retain antioxidant and anti-inflammatory activities when delivered within PDEVs, contributing directly to observed therapeutic effects [5,70,92,96].
- Proteins: while generally present at lower abundance than nucleic acids or metabolites, vesicle-associated proteins, e.g., transporters, chaperones, can influence structural integrity and cellular uptake pathways [7,16,91].

Carrier functions arising from vesicle structure consider:

- Protection of cargo: the lipid bilayer shields encapsulated molecules from enzymatic degradation in biological fluids, particularly in the GI tract during oral administration [8,9,95].
- Enhanced uptake and targeting: natural membrane composition facilitates cellular internalization via endocytic pathways and can confer tropism toward specific cell types, e.g., macrophages or gut epithelium, without synthetic modification [10,11,94].
- Biocompatibility and low immunogenicity: PDEVs are inherently well tolerated, enabling safer systemic delivery relative to many synthetic NPs [5,13,93].

Because many preclinical studies evaluate combined outcomes, e.g., reduced inflammation or tumor suppression, it is often difficult to attribute efficacy solely to intrinsic cargo effects vs. carrier enhancement. To aid conceptual clarity, Table 2 summarizes representative examples from the literature that illustrate distinct contributions of intrinsic molecular cargo vs. carrier-mediated delivery functions.

This delineation underscores that PDEV-mediated therapeutic outcomes often reflect interactions between intrinsic bioactive cargo and structural delivery functions. While endogenous components such as miRNAs and phytochemical metabolites can directly modulate mammalian biological processes, the vesicular membrane concurrently promotes protection and efficient delivery. Distinguishing these roles through controlled mechanistic studies, e.g., cargo depletion, multi-omics profiling, and dose–response separation of endogenous vs. exogenous cargo, will be essential for advancing PDEVs as multifunctional therapeutic nanoplatforms rather than simply passive carriers.

### 2.5. Encapsulation Efficiency and Pharmacokinetic Modulation

Multiple plant-derived platforms demonstrate improved encapsulation efficiency for hydrophobic compounds and enhanced systemic exposure in preclinical models [14,20,48,66,71,72,73,96,97,98,99,100].

Commercial phytosome formulations, particularly curcumin and silymarin phospholipid complexes, have shown increased oral bioavailability relative to unformulated compounds in human PK studies [8,37,38,42,43,44,45,101]. Lipid-based systems similarly enhance solubilization and absorption profiles in experimental settings [3,5,48,49,50,59,60].

These findings support continued evaluation of plant-derived carriers as strategies for bioavailability enhancement, while recognizing that improved PK does not necessarily equate to confirmed clinical efficacy.

### 2.6. Formulation Flexibility

Plant-based nanocarriers can be engineered with targeting ligands, surface coatings, inorganic components, or polymeric matrices to modulate release kinetics and biodistribution [3,5,8,13,14,20,37,38,39,40,41,42,43,44,45,48,49,50,58,59,60,65,66,67,68,69,70,71,72,73,96,97,98,99,100,101].

This structural adaptability supports experimental application across oral, dermal, mucosal, and parenteral routes. Comparative head-to-head benchmarking across platforms remains limited.

### 2.7. Clinical Status

Human evidence remains confined largely to early-phase or PK-focused studies involving selected phytosome formulations and exploratory plant-derived vesicle preparations [18,19,37,38,39,62,102,103,104].

Available data indicate acceptable short-term tolerability and improved systemic exposure for certain compounds. However, adequately powered randomized efficacy trials and long-term safety studies are still lacking.

### 2.8. Alignment with Sustainable Therapeutic Development

Plant-derived delivery systems are increasingly investigated within broader efforts to develop renewable, lower-impact manufacturing strategies and biologically compatible therapeutic platforms [4,15,89,105].

Their long-term translational relevance will depend on validated scalability, standardized production, mechanistic clarity, and regulatory alignment.

Plant-based nanocarriers constitute a heterogeneous but evolving class of delivery systems characterized by renewable sourcing, compositional diversity, and preclinical evidence of favorable tolerability and PK enhancement. While promising, their clinical utility requires confirmation through standardized manufacturing frameworks, mechanistic validation, and rigorously designed human studies.

## 3. Development and Diversity of Plant-Based Carrier Systems

### 3.1. Sources and System Diversity

Plant-based delivery systems include both naturally secreted PDEVs and engineered formulations derived from plant metabolites or extracts. These encompass phytosomes, NLCs, SLNPs, nanoemulsions, mesoporous silica hybrids, green synthesized metal NPs, hybrid inorganic–organic constructs, and PBNPs [1,2,8,9,15,16,36,51,52,53,76,77,78,89,98,106,107,108,109,110,111,112,113,114,115,116,117,118,119,120,121,122,123,124,125,126,127,128,129,130,131,132,133,134,135].

PDEVs contain proteins, lipids, nucleic acids, and small metabolites and have been investigated for roles in intercellular signaling and potential cross-kingdom interactions [4,7,8,9,10,11,12,13,14,15,16,17,18,19,20,22,23,24,25,26,27,28,29,30,31,32,33,48,74,75,76,77,78,89,90,96,97,98,99,100,101,102,103,104,106,110,111,112,113,114,115,116,117,118,119,120,121,122,123,124,125,126,127,128,129,130,131,132,133,134,135,136,137,138,139,140,141]. Engineered phytocarriers use plant-derived molecules as active ingredients, stabilizers, or reducing agents to enhance solubility, stability, and delivery efficiency.

#### 3.1.1. Edible Botanical Sources

Common edible plants, including *Zingiber officinale*, *Citrus* × *paradisi*, *Citrus limon*, *Vitis vinifera*, *Malus domestica*, and *Brassica oleracea*, have been reported as sources of vesicles or phytocarrier materials in experimental studies [4,7,9,11,13,14,22,25,97,101,123,132,139]. Their established dietary exposure may facilitate translational investigation, although regulatory evaluation remains formulation-specific.

#### 3.1.2. Medicinal Plant Families as Phytochemical Reservoirs

Several plant families provide chemically diverse metabolites incorporated into carrier systems such as (*i*) *Zingiberaceae*—curcuminoids and gingerols investigated for anti-inflammatory applications (e.g., *C. longa*, *Z. officinale*) [13,14,15,16,60,97,142]; (*ii*) *Araliaceae*—ginsenosides from *Panax ginseng* studied for metabolic and immunomodulatory effects [4,19,20,38,98]; (*iii*) *Asteraceae*—silymarin from *S. marianum* and related flavonoids evaluated for hepatoprotective and immunomodulatory properties [34,35,36,48,77,136]; (*iv*) *Lamiaceae*—flavonoids and terpenoids explored for antimicrobial and neuroprotective activity [13,28,29,30,48,51,58]; and (*v*) *Meliaceae*—limonoid-rich extracts from *Azadirachta indica* utilized in green NP synthesis strategies [33,42,54,55,56,57].

These families provide structurally diverse phytochemical scaffolds that may influence physicochemical behavior, matrix interactions, and biological responses. Standardized comparative evaluation is needed to determine their relative translational value.

### 3.2. Integrative Overview of Plant-Based Carrier Platforms

Many plant-derived bioactive compounds demonstrate pharmacological activity but face translational constraints due to limited aqueous solubility, chemical instability, rapid metabolism, or suboptimal tissue exposure. To address these limitations, multiple plant-based carrier platforms have been developed, including PDEVs, phytosomes, NLCs, SLNPs, PBNPs, hybrid inorganic–organic systems, and green-synthesized metal/metal oxide NPs.

To consolidate fragmented evidence across botanical origin, phytochemical composition, carrier architecture, biological activity, and therapeutic relevance, Table 3 provides a species-focused integrated mapping of medicinal plants, their key metabolites, associated carrier systems, reported bioactivities (intrinsic and carrier-modulated), and potential translational applications. This overview emphasizes botanical diversity and facilitates rational selection of carrier platforms based on phytochemical reservoirs.

### 3.3. Comparative Evaluation of Plant-Derived Nanocarrier Classes

Given the diversity of plant-based carrier systems, comparative evaluation across translational parameters is necessary to contextualize their development status. Key criteria include scalability (production yield and reproducibility), manufacturing cost, therapeutic performance (bioavailability enhancement, biological activity, targeting potential), safety/biocompatibility, and regulatory or clinical maturity.

Building on the species-specific examples in Table 3, Table 4 provides a platform-centric comparative evaluation of major plant-derived nanocarrier classes across key translational parameters: scalability, manufacturing cost, therapeutic performance, safety/biocompatibility, and clinical/regulatory readiness. The scoring (1–5) represents a qualitative synthesis of recent literature, preclinical studies, and early clinical evidence from 2023 to 2026, serving as relative positioning indicators rather than absolute performance metrics.

Table 3 and Table 4 are complementary in scope and purpose. Table 3 highlights species-level diversity by summarizing representative plant sources, associated phytochemicals, and corresponding nanocarrier systems. In contrast, Table 4 provides a platform-centric comparison, benchmarking major plant-derived nanocarrier classes across key translational attributes (e.g., scalability, cost, therapeutic performance, safety, and clinical readiness) to facilitate rational platform selection.

### 3.4. Limitations in Direct Cross-Platform Comparisons and Proposed Standardized Metrics

Although the preceding sections provide a comprehensive overview of plant-derived nanocarrier platforms, including PDEVs, phytosomes, lipid-based NPs, polymeric carriers, and self-assembled phytochemical systems, it is important to recognize that direct cross-platform comparisons conducted under standardized experimental conditions remain scarce. Most studies in this field focus on the development and characterization of individual delivery systems and are typically performed using heterogeneous experimental frameworks that vary in plant sources, encapsulated phytochemical cargos, formulation strategies, analytical methods, cellular models, and animal models. Consequently, direct benchmarking across platforms, particularly for translational parameters such as oral bioavailability, cellular uptake efficiency, PK performance, and biodistribution profiles, remains limited. Recent reviews emphasize that this methodological diversity represents a major barrier to systematic evaluation of plant-derived nanocarriers and complicates attempts to identify universally superior delivery platforms [5,91,92].

Among emerging delivery systems, PDEVs have attracted considerable attention as naturally occurring nanoscale carriers composed of lipid bilayer membranes enriched in plant lipids, proteins, and nucleic acids. These vesicles demonstrate promising biological compatibility and have shown stability in simulated GI environments, along with efficient uptake by intestinal epithelial cells and immune cells [9,10,11]. For example, ginger-derived PDEVs have been reported to exhibit robust internalization by macrophage cell lines such as RAW264.7, suggesting their potential utility for targeted immunomodulatory delivery [12,148]. Although such findings highlight the unique biological properties of PDEVs, most available studies evaluate single-vesicle systems derived from specific plant species, and comparative analyses with alternative nanocarrier classes under identical experimental conditions remain uncommon [8,77,99].

In contrast, phytosomes and related phospholipid–phytochemical complexes represent one of the most mature nanocarrier technologies developed for improving the delivery of plant-derived bioactive compounds. These systems typically involve the molecular complexation of phytochemicals with phospholipids such as PC, resulting in enhanced membrane permeability and improved GI absorption. Numerous PK studies have demonstrated significant increases in oral bioavailability for poorly soluble phytochemicals, including curcumin and silybin, with reported improvements often ranging between 5- and 20-fold relative to the free compounds [37,38]. As an illustrative example, curcumin phytosome formulations have demonstrated enhanced lymphatic transport and improved systemic exposure in rodent PK models, supporting their clinical translational potential. However, despite this PK maturity, direct experimental comparisons between phytosomes and emerging natural nanocarriers such as PDEVs remain rare, limiting the ability to quantitatively assess their relative performance.

Similarly, lipid-based NPs, including solid lipid nanoparticles (SLNs) and nanostructured lipid carriers (NLCs), have been widely explored as delivery systems for lipophilic phytochemicals. NLCs in particular frequently demonstrate improved loading capacity and controlled release compared with SLNs because their hybrid solid–liquid lipid matrices generate structural imperfections that facilitate drug incorporation [92,95]. Nevertheless, comparisons between lipid NPs and other plant-derived nanocarriers remain complicated by differences in experimental design, including the use of distinct model compounds, cell lines, and dosing protocols.

Additional strategies include polymeric or protein-based plant-derived NPs, such as carriers based on zein, chitosan, or other plant biopolymers. These materials may offer advantages such as mucoadhesion, prolonged GI residence time, and potential colon-targeted delivery of phytochemicals and nutraceuticals [94]. Furthermore, self-assembled phytochemical nanostructures and hybrid plant-inspired nanocarriers have emerged as alternative approaches capable of improving the solubility and stability of hydrophobic plant bioactives [93]. However, the diversity of formulation strategies and evaluation models used in these studies further underscores the need for harmonized experimental criteria that would enable more rigorous cross-platform comparisons.

To provide a structured overview of the principal nanocarrier systems discussed in this review, Table 5 summarizes the main physicochemical and translational attributes of representative plant-derived nanocarrier platforms reported in the literature. Because standardized head-to-head experimental comparisons remain limited, the table synthesizes commonly reported characteristics and evaluation models rather than presenting direct quantitative benchmarking.

Beyond descriptive comparison, the field would benefit substantially from harmonized experimental frameworks that enable quantitative cross-platform benchmarking. The absence of standardized evaluation protocols currently limits the ability to directly compare PK performance, cellular uptake, and biodistribution across nanocarrier systems. To address this gap, Table 6 proposes a standardized evaluation framework that could facilitate more rigorous comparative studies in future research. Such benchmarking approaches could allow researchers to identify platform-specific advantages while improving reproducibility and translational relevance.

Overall, the implementation of standardized evaluation strategies would facilitate more robust comparisons among plant-derived nanocarrier platforms and help clarify their respective advantages for phytochemical delivery. Until such datasets become more widely available, interpretation of nanocarrier performance should rely on integrated analysis of physicochemical properties, biological interactions, and translational potential rather than direct cross-platform ranking.

## 4. Isolation and Purification of Plant-Derived Extracellular Vesicles

The isolation and purification of PDEVs, also known as PELNPs or nanovesicles, is a critical step in developing plant-based delivery platforms [7,10,19,22,24,25,26,27,28,29,112]. These processes determine vesicle yield, purity, structural integrity, cargo preservation, and downstream applicability. PDEVs (30–500 nm) are secreted into the apoplast, root exudates, xylem/phloem fluids, or culture media, carrying bioactive cargos, including lipids, proteins, microRNAs, and secondary metabolites, that mediate cross-kingdom biological effects. Efficient isolation requires removal of debris, protein aggregates, organelle fragments, and cell wall polysaccharides while maintaining vesicle functionality, a challenge compounded by rigid cellulose- and pectin-rich cell walls, tissue-specific heterogeneity, and vesicle subpopulation diversity (size, density 1.10–1.18 g/mL, cargo profile) [7,10,19,22,24,25,26,27,28,29,112].

Between 2020 and 2026, methodological advances have emphasized scalability, purity, recovery, and GMP compliance, yielding up to 10^12^–10^13^ vesicles per gram of tissue or liter of culture medium from edible and medicinal plants. Representative sources include fruit juices (citrus, apple, pomegranate), vegetable homogenates (broccoli, tomato), herbal infusions (green tea, ginseng), apoplast washing fluid (AWF), and root exudates. Pre-treatments, such as enzymatic digestion or sonication, facilitate vesicle release. Combinatorial isolation strategies, such as DUC, SEC, ultrafiltration (UF)/TFF, are often required, as no single method consistently optimizes yield, purity, and structural integrity.

### 4.1. Key Isolation Methods

#### 4.1.1. Differential Ultracentrifugation

DUC remains standard for volumetric throughput and size/density stratification (50–500 nm). Sequential centrifugation removes cells, debris, and organelles, followed by high-speed ultracentrifugation (100,000–150,000× *g*) to isolate PDEVs. Advantages include scalability, cost-effectiveness, and suitability for large samples. Limitations refer to moderate recovery (30–60%), shear-induced damage, and co-isolation of proteins, requiring downstream purification. Ginsenoside-rich PDEVs from *P. ginseng* and alkylamide-loaded vesicles from *Echinacea purpurea* are the main applications [3,4,5,11,18,19,26,27,28,32,33,34,35,36,37,38,39,40,41,42,43,44,45,47,51,52,53,61,62,63].

#### 4.1.2. Density Gradient Ultracentrifugation

Density gradient ultracentrifugation (DGUC) separates vesicles based on buoyant density (1.10–1.18 g/mL), often using iodixanol gradients. Advantages include high purity (>95%) and subpopulation isolation; limitations are lower throughput and potential bioactivity loss with certain gradients. The method is effective for polyphenol-rich PDEVs from *C. sinensis*, *Matricaria chamomilla*, tomato, and citrus, enabling antioxidant, anti-inflammatory, and chemosensitizing effects [3,4,5,11,18,19,26,27,28].

#### 4.1.3. Size-Exclusion Chromatography

SEC separates PDEVs based on size through porous media. Advantages refer to rapid processing, minimal shear, high recovery (70–90%); limited sample capacity and potential co-elution of similar-sized contaminants are the main limitations. Peppermint, rosemary, pomegranate, and cabbage-derived vesicles are obtained using this method [3,4,5,11,18,19,22,25,26,27,28,32,33,34,35,36,37,38,39,40,41,42,43,44,45,47,51,52,53,61,62,63].

#### 4.1.4. Polyethylene Glycol Precipitation

Polyethylene glycol (PEG) induces vesicle aggregation, followed by low-speed centrifugation. Advantages include simplicity and scalability; co-precipitation of proteins/nucleic acids and potential subpopulation bias are considered as limitations. This isolation technique is used for clove, cinnamon, and *Solanum nigrum* PDEVs [26,27,28,31].

#### 4.1.5. Ultrafiltration and Tangential Flow Filtration

Membrane filtration separates vesicles by size (0.1–0.8 μm, 100 kDa cutoff). Advantages refer to high recovery and gentle handling, and limitations include membrane adsorption and clogging. The method is applied to *Nelumbo nucifera*, cabbage, and hybrid PDEV–inorganic carriers [32,33,34,38,43,44,45].

#### 4.1.6. Enzymatic Digestion and Ultrasound-Assisted Extraction

Cellulase/pectinase digestion and ultrasound enhance vesicle release from fibrous tissues. Combined approaches facilitate hybrid carrier assembly, e.g., phytochemical immobilization onto mesoporous silica, halloysite nanotubes (HNTs), or metallic NPs [40,41,42,43,44,61,62,63].

#### 4.1.7. Immunoaffinity Capture and Emerging Techniques

Immunoaffinity (IA) capture uses biomarker-specific antibodies (e.g., anti-TET8) for subclass isolation with high specificity but low yield [47]. Emerging strategies include microfluidics, acoustic separation, and artificial intelligence (AI)-guided combinatorial pipelines, supporting GMP-compliant production [32,33,34,38,40,41,42,43,44,45,47,51,52,53,61,62,63].

#### 4.1.8. Comparative Overview

To support the selection of appropriate PDEV isolation methods, Table 7 summarizes the principal techniques, highlighting their working principles, advantages, limitations, representative plant applications, and recent methodological innovations. This comparative overview aids researchers in choosing scalable, high-purity, and cargo-preserving strategies suitable for different plant sources and downstream applications.

### 4.2. Challenges and Integrated Strategies

Persistent challenges of the isolation and purification techniques of PDEVs include (*i*) biological heterogeneity—seasonal, tissue-specific, and environmental variability affect vesicle size, *zeta* potential, and cargo composition; (*ii*) low yields from fibrous tissues—roots and bark yield <10^10^ vesicles/g without pre-treatment; (*iii*) downstream processing limitations—centrifugation, filtration, and TFF can cause shear stress and adsorption losses; (*iv*) regulatory uncertainty—no unified U.S. Food and Drug Administration (FDA)/European Medicines Agency (EMA) guidance, the products can be classified as biologics, botanicals, or nanomaterials; and (*v*) incomplete mechanistic and safety data—human uptake, endosomal escape, and chronic dosing remain understudied. Mitigation strategies include multi-step isolation pipelines (DUC, SEC and TFF), enzymatic/ultrasound pre-treatment, predictive machine learning (ML) for process optimization, and hybrid engineering with inorganic matrices to improve stability and multifunctionality [7,10,19,22,24,25,26,27,28,29,112].

### 4.3. Future Perspectives

Key areas for translational advancement of the isolation and purification techniques of PDEVs pursue main objectives such as (*i*) AI/ML-driven design for predictive optimization of source plants, cargo loading, and purification for reproducible vesicles; (*ii*) controlled cultivation and bioreactors using continuous, GMP-compliant production with renewable biomass and valorized residues; (*iii*) hybrid and stimuli-responsive carriers for integration with mesoporous silica, Fe_3_O_4_, HNTs, or polymers for targeted, triggered release; (*iv*) RNA and precision therapeutics in case of plant-derived vesicles for small interfering RNA (siRNA)/messenger RNA (mRNA) delivery coupled with patient stratification; and (*v*) regulatory engagement for standardized quality control, Minimal Information for Studies of Extracellular Vesicles (MISEV)-guided characterization, and early-phase clinical evaluation. Plant-based carriers, including PDEVs, phytocarriers, and hybrid PBNPs, are positioned as sustainable, immunologically compatible, and multifunctional platforms for oral, targeted, and RNA-based therapies [7,10,19,22,24,25,26,27,28,29,112,123,124].

## 5. Engineering and Functionalization Strategies

The engineering and functionalization of PDEVs, together with advanced phytocarriers (phytosomes, NLCs, SLNPs, and nanoemulsions) and PBNPs, mark a critical transition from naturally bioactive vesicles to programmable nanomedicine platforms. Although native PDEVs possess intrinsic therapeutic properties, rational bioengineering is essential to optimize drug loading efficiency, targeting specificity, PK, and manufacturing reproducibility for clinical translation [75,133,154].

PDEVs are nanoscale lipid bilayer vesicles (30–500 nm) enriched in phospholipids, sterols, membrane-associated proteins, miRNAs, and plant-derived secondary metabolites. They exhibit biocompatibility, low immunogenicity, GI stability, and cross-kingdom communication mediated by conserved endocytic and receptor-dependent uptake pathways in mammalian systems [14,75,155,156]. Complementary phytocarriers, including phytosomes, SLNPs, and NLCs, enhance solubility and systemic bioavailability of poorly water-soluble phytochemicals, whereas PBNPs assembled from plant polysaccharides, lipids, or proteins provide tunable physicochemical properties and controlled release profiles [34,35,36,37,38,39,40,41,42,43,44,45,46,47,57,58,59,60,106,107,113,114,115,116,117,157].

Despite these advantages, native PDEVs face translational constraints, including moderate cargo loading efficiency, limited targeting precision, variable cargo stability, and batch-to-batch heterogeneity. Between 2020 and 2026, advances in EVs bioengineering, surface chemistry, and hybrid nanotechnology have enabled scalable loading strategies, ligand-directed targeting, and multifunctional hybrid constructs [75,133,134,154].

Engineering strategies can be categorized into three principal domains: cargo loading strategies, surface functionalization approaches, and hybrid engineering platforms.

### 5.1. Cargo Loading Strategies

Efficient cargo incorporation is fundamental for translational applicability. Methods originally developed for mammalian EVs have been adapted to plant-derived systems, with careful optimization to preserve membrane integrity and biological functionality [75,133].

#### 5.1.1. Passive Loading

Passive loading relies on diffusion-driven partitioning of therapeutic agents into vesicle membranes or luminal compartments during co-incubation. Encapsulation efficiencies typically range from 30 to 70%, depending on cargo hydrophobicity, lipid composition, incubation conditions, and buffer parameters [75,133].

Applications include small-molecule chemotherapeutics (e.g., doxorubicin (DOX)), siRNA, miRNA mimics, and hydrophobic phytochemicals. Passive loading preserves vesicle integrity and is technically straightforward, supporting scalability. Limitations refer to lower loading efficiency and potential post-loading leakage [75,134].

#### 5.1.2. Active Loading

Active strategies such as electroporation, sonication and freeze–thaw cycles and extrusion transiently increase membrane permeability to enhance cargo incorporation.

Electroporation applies short electrical pulses to induce temporary membrane nanopores, facilitating the uptake of nucleic acids and small molecules. Efficiencies of 60–90% have been reported, though aggregation may occur under non-optimized conditions [75,133].

Through sonication, mechanical shear temporarily disrupts membrane organization, promoting hydrophobic drug incorporation. Excessive sonication may alter vesicle morphology [75,154].

Freeze–thaw cycles and extrusion use repeated cycles or membrane extrusion to promote cargo entrapment, particularly in hybrid or reconstituted vesicles [133,134].

Loading efficiency depends on lipid composition, surface charge (−10 to −35 mV), cargo molecular weight and polarity, and buffer ionic strength. Standardization is critical for reproducibility and regulatory compliance [74,123].

Recent advances in EV engineering have substantially expanded the therapeutic versatility of vesicle-based delivery systems. In particular, engineered EV platforms capable of efficient intracellular transport of biologically active proteins without requiring genetic fusion tags have demonstrated markedly improved cytosolic and nuclear delivery efficiencies in preclinical models [158]. These approaches exploit membrane remodeling, cargo-loading optimization, and vesicle surface engineering to facilitate enhanced intracellular trafficking and functional payload release.

Although initially developed primarily in mammalian EV systems, the underlying design principles, including controlled cargo encapsulation, modulation of membrane permeability, and the incorporation of targeting ligands, are increasingly being explored in PDEVs and exosome-like NPs isolated from edible or medicinal plants. A growing body of evidence indicates that PDEVs possess intrinsic biocompatibility, natural bioactive cargo, and the ability to participate in cross-kingdom molecular communication, enabling the delivery of plant-derived RNAs, proteins, and metabolites to mammalian cells [11,14,61,62].

Recent studies have further highlighted the therapeutic potential of PDEVs as natural nanocarriers for drug delivery in cancer therapy, regenerative medicine, and neurological disorders, where they can modulate cellular signaling pathways while maintaining favorable safety profiles [12,15,18,20]. Advances in isolation technologies, vesicle characterization, and scalable production strategies are also contributing to improved reproducibility and translational feasibility of plant-derived vesicle systems [7,22,27,30].

Within the broader context of plant-based nanotherapeutics, PDEVs complement other phytogenic nanocarrier platforms, including phytosomes, plant-synthesized metal NPs, and lipid-based phyto-nanostructures, which have demonstrated promising potential for enhancing the bioavailability and therapeutic efficacy of plant-derived bioactive compounds [35,36,41,55,57].

Consequently, the integration of vesicle engineering strategies with plant-derived nanocarrier platforms provides a compelling conceptual framework for the development of next-generation engineered PDEVs capable of delivering complex therapeutic cargos, including proteins, nucleic acids, and bioactive phytochemicals, with improved targeting efficiency and translational potential.

### 5.2. Surface Functionalization for Targeted Delivery

Surface modification improves biodistribution, receptor-mediated uptake, and therapeutic specificity. Chemical conjugation approaches, e.g., carbodiimide-mediated coupling (1-ethyl-3-(3-dimethylaminopropyl)carbodiimide (EDC)/*N*-hydroxysuccinimide (NHS)), lipid-anchor insertion, click chemistry, and non-covalent adsorption, have been successfully adapted for plant vesicles [75,144,145]. Grapefruit-derived vesicles serve as a well-characterized proof-of-concept, demonstrating stable ligand conjugation without compromising vesicle integrity or bioactivity [144,145].

#### 5.2.1. Peptide-Based Targeting

Cyclic arginine–glycine–aspartate (cRGD) peptides selectively bind integrin αvβ3 receptors in tumor vasculature, enhancing tumor accumulation and cellular uptake in preclinical oncology models [133,154].

#### 5.2.2. Aptamer Functionalization

Aptamer-conjugated vesicles, e.g., AS1411 targeting nucleolin, enable selective intracellular delivery of siRNA and chemotherapeutics. High binding affinity with minimal immunogenicity supports their application in RNA therapeutics [75,133].

#### 5.2.3. Antibody and Ligand Conjugation

Ab- or ligand-mediated strategies enhance tumor selectivity and therapeutic index. Preclinical studies show improved receptor-mediated internalization and reduced off-target effects [133,134,154].

Surface-functionalized PDEVs improve in vivo targeting and therapeutic amplification; however, reproducibility and regulatory standardization remain challenges [123,128].

### 5.3. Hybrid Engineering and Advanced Constructs

Hybrid engineering combines PDEVs with synthetic lipids, polymers, or inorganic nanomaterials to introduce functionalities beyond native properties [58,59,60,113,114,115,116,117,133,142].

#### 5.3.1. Lipid Fusion and PEGylation

Fusion with synthetic lipid bilayers, including PEGylated phospholipids, prolongs circulation time, enhances colloidal stability, and enables sustained drug release [75,133].

#### 5.3.2. Polymer-Coated Systems

Polymeric coatings such as PEG or poly(lactic-*co*-glycolic acid) (PLGA) enhance stability, reduce aggregation, and permit stimuli-responsive release (pH- or redox-sensitive) [75,134].

#### 5.3.3. Inorganic–Organic Hybrid Platforms

PDEVs and phytochemicals have been integrated into multifunctional platforms with mesoporous silica, Fe_3_O_4_, Au, Ag, and kaolinite. These hybrids enable combined therapeutic and diagnostic applications (theranostics), leveraging PDEV biocompatibility and inorganic structural robustness [3,4,5,47,48,58,59,60,107,109,110,113,114,115,116,117,157,159].

Hybrid engineering strategies have emerged as powerful approaches for enhancing vesicle functionality and expanding the therapeutic capabilities of bioinspired nanocarriers. Increasing evidence indicates that engineered EVs represent a versatile class of nanomedicine platforms capable of integrating targeting ligands, multifunctional therapeutic payloads, and stimuli-responsive elements to achieve improved PK behavior and controlled drug delivery [160]. Complementary developments in peptide–lipid hybrid vesicle systems demonstrate how rational membrane design can introduce stimuli-responsive phase separation within vesicular membranes, enabling dynamic modulation of membrane organization and triggered release of encapsulated cargos under specific physiological conditions [161]. Such approaches provide valuable design principles for the development of advanced phyto-hybrid nanocarriers, in which plant-derived vesicles may be combined with synthetic lipids, peptides, or polymers to enhance structural stability, targeting precision, and therapeutic performance.

### 5.4. Comparative Overview

Table 8 provides a comprehensive overview of engineering strategies for PDEVs and hybrid plant-based nanocarriers, integrating methods for cargo loading, surface functionalization, and hybridization. By explicitly linking methodology to functional enhancements, including encapsulation efficiency, targeting specificity, circulation stability, and multifunctionality, it underscores how rational design translates into improved therapeutic performance. This complements physicochemical characterization and in vitro/in vivo evaluations, providing readers with a practical framework for selecting engineering strategies to optimize pharmacological outcomes.

### 5.5. Challenges and Future Perspectives

Despite rapid progress, several scientific, technical, manufacturing, regulatory, and translational challenges impede clinical adoption.

Key limitations include (*i*) cargo-induced instability—active loading may destabilize vesicles and cause leakage, and passive loading often provides suboptimal encapsulation; (*ii*) biological heterogeneity induced by seasonal, genotype, and tissue-specific variations, affecting vesicle composition and function, and complicating standardization; (*iii*) scalability—current isolation techniques are low throughput, labor-intensive, and difficult to scale without compromising integrity; (*iv*) regulatory uncertainty because PDEVs are variably considered biologics, botanical drugs, or nanomaterials, requiring case-by-case documentation [123,124,128,138]; and (*v*) mechanistic gaps due to endosomal escape, functional cargo delivery, off-target effects, and long-term safety of hybrid systems remain understudied [14,89,96,149].

Multi-step isolation pipelines, predictive process optimization and hybrid engineering platforms are the most valuable mitigation strategies. Multi-step isolation pipelines, e.g., DUC–SEC–TFF, or enzymatic digestion–microfiltration/sonication, can improve purity, reproducibility, and functional consistency [22,24,25,29]. Predictive process optimization using ML and integrative omics can guide loading, surface modification, and formulation [34,97]. Hybrid engineering platforms combining PDEV scaffolds with lipids, polymers, or inorganic matrices can enhance stability, targeting, and multifunctionality [58,59,60,109,133,134].

Future directions (toward 2030) are focused on (*i*) AI-driven predictive design integrating physicochemical and multi-omics datasets for optimized vesicle architectures and hybrid constructs [34,97]; (*ii*) scalable GMP-compliant production using perfusion bioreactors, hollow fiber platforms, and temporary immersion systems, including valorization of plant residues [31,32,152]; (*iii*) smart, stimuli-responsive hybrids for pH/redox/enzyme-triggered release, magnetic guidance, and combinatorial therapies [58,59,60,103,104,105,106,107,108,109,110,111,112,113,114,115,116,117,133,134,142]; (*iv*) RNA therapeutics and precision delivery with patient stratification for cancer, autoimmune, and metabolic disorders [75,133,162]; and (*v*) expanded clinical pipeline and regulatory engagement, leveraging early human and veterinary safety data to advance pivotal trials by 2028–2030 [97,128].

Plant-based nanocarriers represent sustainable, immunologically privileged, and cost-efficient alternatives for targeted, oral, RNA-based, and regenerative therapies, harmonizing therapeutic innovation with ecological stewardship and global health priorities [8,97,128,142].

## 6. Characterization of Plant-Based Carrier Systems

### 6.1. Overview and Importance

Rigorous characterization of plant-based carrier systems, including PDEVs and PBNPs, is essential to define identity, purity, structural integrity, compositional profiles, stability, and functional properties. Comprehensive physicochemical and multi-omics profiling ensures reproducibility, inter-laboratory comparability, mechanistic elucidation, and optimized drug-loading or targeting strategies, while supporting regulatory compliance for preclinical and translational development. These principles align with adapted MISEV2023 guidelines released by the International Society for Extracellular Vesicles (ISEV) and emerging plant-specific standards emphasizing orthogonal characterization and minimal information reporting [8,9,74,123,124].

PDEVs are distinguished by a lipid bilayer encapsulating heterogeneous cargo (lipids, proteins, nucleic acids, and metabolites), while PBNPs often exhibit engineered architectures derived from plant polysaccharides, proteins, or lipids, with tunable surface chemistry and controlled release properties. Integration of multi-omics approaches (proteomics, lipidomics, small RNA sequencing, metabolomics) with advanced biophysical techniques enables species-specific profiling, revealing molecular signatures associated with medicinal plants and addressing cargo heterogeneity and co-isolated contaminants [8,9,12,13,14,102].

### 6.2. Physicochemical Properties and Analytical Techniques

Physicochemical characterization provides quantitative metrics, including size, surface charge, particle concentration, morphology, and mechanical properties, that correlate with colloidal stability, cellular uptake, biodistribution, and in vivo performance.

#### 6.2.1. Hydrodynamic Size, Surface Charge, and Particle Concentration

Dynamic light scattering (DLS) and nanoparticle tracking analysis (NTA) measure hydrodynamic diameter, polydispersity index (PDI), *zeta* potential, and particle concentration. PDEVs typically range from 50 to 500 nm, with major subpopulations between 100 and 300 nm. A PDI < 0.3 indicates monodispersity. *Zeta* potentials of −10 to −35 mV confer electrostatic stabilization, as reported for ginger, grapefruit, and ginseng-derived carriers [8,9,12,102].

NTA provides single-particle resolution without assuming spherical geometry, while DLS offers rapid ensemble averages; combining both enhances robustness and reproducibility [74,124].

Negative *zeta* potentials result from surface phospholipids and proteins, contributing to the colloidal stability in physiological media, as seen in *Ginkgo biloba* ginkgolide–PDEVs (−20 to −30 mV) and *Valeriana officinalis* valerenic acid–PDEVs [102,123].

#### 6.2.2. Morphology and Structural Integrity

Transmission electron microscopy (TEM) and cryo-TEM confirm classical cup-shaped morphology, lipid bilayer thickness (~4–7 nm), and absence of aggregates or contaminants. Cryo-TEM preserves native hydrated states and reveals internal cargo, as well as engineered nanocarrier architectures such as NLCs, SLNPs, and mesoporous silica hybrids [10,12,13,102].

Scanning electron microscopy (SEM) complements TEM for surface morphology, especially in hybrid or inorganic–organic carriers [47,58,59,60,113,114,115,116,117,142].

#### 6.2.3. Surface Topography and Mechanical Properties

Atomic force microscopy (AFM) provides high-resolution maps of surface topography, stiffness, roughness, and adhesion, critical for hybrid PBNPs where surface features influence cellular interactions and formulation stability [15,16,102]. AFM also evaluates membrane fluidity, elasticity, and resilience, relevant to GI transit and cellular uptake [8,12].

#### 6.2.4. Complementary Orthogonal Techniques

Complementary orthogonal techniques include (*i*) tunable resistive pulse sensing (TRPS) for single-particle size, charge, and concentration [24]; (*ii*) flow cytometry applying fluorescent labeling (e.g., PKH26, DiI) for subpopulation heterogeneity and uptake dynamics [25]; (*iii*) small-angle X-ray scattering (SAXS) and small-angle neutron scattering (SANS) for low-resolution three-dimensional (3D) structural information in solution [74]; and (*iv*) Raman and Fourier-transform infrared (FTIR) spectroscopy for molecular fingerprinting of lipid–phytochemical interfaces [20,102]. Integration of these orthogonal measurements ensures mechanistic understanding, reproducible formulation, and regulatory-aligned documentation, establishing plant-based carriers as translationally promising platforms [8,9,10,12,13,15,16,74,102,123,124].

The increasing recognition of EV heterogeneity has stimulated the development of advanced analytical platforms capable of characterizing vesicles at the single-particle level. Single-EV analysis technologies, including high-resolution imaging, label-free detection systems, and high-throughput analytical methods, allow detailed investigation of vesicle size distribution, molecular cargo heterogeneity, and functional variability across vesicle populations. These approaches have become particularly valuable in biomedical research for elucidating EV-mediated signaling mechanisms and therapeutic delivery pathways, especially in oncology applications [163,164]. The application of these methodologies to plant-derived vesicles is expected to significantly enhance the molecular characterization, cargo profiling, and functional assessment of PDEVs, thereby supporting the establishment of standardized analytical frameworks necessary for reproducible manufacturing, quality control, and regulatory evaluation of plant-based nanocarrier systems.

#### 6.2.5. Compositional Analysis and Cargo Profiling

Multi-omics analyses elucidate the molecular cargo responsible for intrinsic bioactivity and drug delivery potential in PDEVs, engineered phytocarriers, and PBNPs [16,20,26,27,33,36,37,38,39,40,41,42,43,44,45,46,47,55,57,63,64,65,66,67,68,69,70,71,72,73,113,114,115,116,117,157,159]. These studies reveal endogenous components that synergize with therapeutics, enabling dual functionality (carrier–bioactive molecule), supporting quality control, batch standardization, and regulatory submissions [8,9,12,13,47,102].

Through liquid chromatography–tandem mass spectrometry (LC–MS/MS), proteomics identifies vesicle-enriched proteins such as membrane-trafficking proteins, chaperones, tetraspanin-like homologs, heat shock proteins, annexins, aquaporins, and Rab guanosine triphosphatases (GTPases), while contaminants (ribulose-1,5-bisphosphate carboxylase/oxygenase (RuBisCO), calnexin, histones, actin, tubulin) remain low [8,12,13]. In hybrid carriers, adsorption of stress proteins enhances structural stability and therapeutic synergy [4,5,58,59].

Lipidomics identify phospholipids (PC, PE, phosphatidylinositol (PI), phosphatidylserine (PS), GIPCs), sterols (stigmasterol, β-sitosterol), and sphingolipids, which confer membrane fluidity, fusogenicity, GI resilience, and signaling properties. Green tea PDEVs, silymarin phytosomes, and hybrid curcumin–silica or silymarin–Fe_3_O_4_ systems exemplify lipid-mediated stability and controlled release [8,9,10,11,12].

Small RNA/nucleic acid profiling highlights miRNAs (e.g., miR-156a, miR-159, miR-396e), long non-coding RNAs (lncRNAs), and siRNAs mediate cross-kingdom gene regulation, anticancer, antiviral, and anti-fibrotic effects. Ginseng PDEVs and *C. longa* hybrids demonstrate miRNA-mediated immunomodulation and neuroprotection [8,14,15,16,17].

Endogenous phytochemicals, e.g., gingerols, shogaols, curcuminoids, ginsenosides, silymarin, quercetin, and punicalagins, are quantified via LC–MS/MS, gas chromatography–mass spectrometry (GC–MS), and nuclear magnetic resonance (NMR) in metabolomics studies. Hybrid carriers enhance synergistic effects, improve efficacy, reduce dosing requirements, and modulate key pathways such as nuclear factor-kappaB (NF-κB), nuclear factor erythroid 2-related factor 2 (Nrf2), and microbiome recalibration [5,8,9,12,24].

These integrated datasets provide a molecular blueprint for functional prediction, standardization, and regulatory submissions, supporting translational pipelines in line with MISEV2023 and ISEV plant-task recommendations.

### 6.3. Stability, Biocompatibility, and Toxicity Evaluations

Robust evaluation of stability, biocompatibility, and toxicity (cytotoxicity, genotoxicity/mutagenicity) confirms translational suitability of plant-based carrier systems.

Stability included pH 1.2–7.4 tolerance, 4–37 °C thermal equilibrium, shear/enzymatic resistance, and serum retention of 10–50% [4,5,6,9,10,18]. Simulated physiological assays show 85–95% integrity for PDEVs and phytocarriers, exceeding conventional liposomes (<60%). Hybrid carriers exhibit further stabilization via inorganic matrices [5,6,18,23,58,59].

Hemolysis < 5%, IL-6/TNF-α/interferon-gamma (IFN-γ) cytokine levels less than baseline, and negligible complement activation are the main parameters for a proper biocompatibility [11,12,19,20,22,23]. In this respect, hybrid carriers such as curcumin–silica, silymarin–Fe_3_O_4_, and quercetin–AuNPs maintain low immunogenicity and excellent tissue compatibility [13,14,15,23,60].

Cytotoxicity is highlighted by a viability > 92% at 150 μg/mL and half-maximal inhibitory concentration (IC_50_) > 500 μg/mL in diverse cell types, superior to cationic NPs [16,17,18,23]. Moreover, Comet assay and micronucleus tests confirm olive tail moment <5% and no mutagenicity [19,20,23].

These evaluations ensure safe, reproducible, and mechanistically understood carriers suitable for therapeutic applications and regulatory approval.

To complement detailed textual descriptions of stability, biocompatibility, and toxicity, Table 9 provides a structured summary of representative plant-based carriers. It consolidates critical experimental metrics, including particle size retention, PDI, hemolysis, cytotoxicity, and genotoxicity, across PDEVs, engineered phytocarriers, and hybrid inorganic–organic NPs. This comparative snapshot facilitates rapid evaluation of physicochemical integrity, safety profiles, and translational suitability, directly supporting mechanistic insights, regulatory considerations, and design optimization of next-generation plant-derived nanotherapeutics.

### 6.4. In Vitro Evaluation: Cellular Uptake and Mechanistic Insights

Plant-based carriers exhibit selective cellular uptake via energy-dependent endocytic pathways (clathrin-mediated, caveolae/lipid-raft, macropinocytosis, receptor-mediated). Fluorescent labeling confirms preferential accumulation in malignant cells 2–4-fold higher than normal fibroblasts, exemplified by *Ocimum basilicum* and *Syzygium aromaticum* PDEVs [6,7,8,9].

Mechanistic effects include miRNA-mediated gene silencing, inflammatory pathway (NF-κB, mitogen-activated protein kinase (MAPK), phosphoinositide 3-kinase/protein kinase B (PI3K/Akt)) modulation, antioxidant activation (Nrf2), apoptosis/autophagy induction, ROS modulation, and antimicrobial activity. Curcumin-loaded turmeric carriers, ashwagandha PBNPs, and hybrid phyto-nanoconstructs demonstrate synergistic bioactivity and intracellular amplification of therapeutics [10,11,12,13,14].

### 6.5. In Vivo Evaluation: Biodistribution, Pharmacokinetic Profile, and Therapeutic Outcomes

Preclinical models demonstrate favorable biodistribution, PK, and therapeutic efficacy of plant-based carrier systems, as follows: (*i*) 30–50% oral bioavailability and 24–72 h extended half-life [15,16,17]; (*ii*) for oncology applications, 55–70% tumor regression with DOX-loaded *Ocimum sanctum* or neem carriers and improving of enhanced permeability and retention (EPR)-mediated tumor localization [18,19]; (*iii*) for GI disorders and immunomodulatory properties, ginger and turmeric carriers reduce inflammatory bowel disease (IBD) activity indices by 50–70%, restore mucosal architecture, and recalibrate microbiota [20,22]; and (*iv*) for neurodegenerative diseases, *M. oleifera* and *V. officinalis* PDEVs cross the blood–brain barrier (BBB), reduce amyloid β deposition, suppress microglial activation, and improve cognition. Also, hybrid magnetic carriers enable guided brain delivery [23,24,25].

GLP-compliant toxicology confirms minimal hepatotoxicity/nephrotoxicity, stable hematological parameters, and low immunogenicity. Histopathology shows no dose-limiting toxicity at therapeutic doses [16,26].

Integration of rigorous physicochemical, multi-omics, stability, biocompatibility, and in vitro/in vivo evaluations establishes plant-based carriers as mechanistically understood, multifunctional nanotherapeutic platforms. Their intrinsic bioactivity, favorable PK/PD profiles, tissue-targeting capacity, microbiome modulation, and safety characteristics position them as next-generation alternatives to synthetic nanocarriers. Standardized characterization, scalable GMP production, and expanded clinical evaluation will be critical to achieving regulatory approval and translational impact [3,4,5,6,15,16,17,18,19,20,22,23,24,25,26].

### 6.6. Translational Status, Safety Considerations, and Barriers to Clinical Application

#### 6.6.1. Translational Landscape

PDEVs together with engineered phytocarriers (e.g., phytosomes, NLCs, SLNPs, nanoemulsions, mesoporous silica–phyto-hybrids, green synthesized metal/metal oxide NPs, and hybrid inorganic–organic phyto-nanoconstructs) and bioinspired PBNPs have demonstrated reproducible biological activity across diverse preclinical models, including oncology, inflammatory disorders, metabolic dysfunction, neurodegeneration, and regenerative applications [3,4,5,6,7,61,126].

These platforms integrate intrinsic phytochemical bioactivity with nanoscale delivery functionality and are commonly characterized, primarily in preclinical systems, by compatibility with oral administration, low observed immunogenicity in controlled models, biodegradability and biological tolerability, renewable plant sourcing, and potential cost advantages relative to mammalian EVs production [3,4,5,6,7,61].

Despite extensive mechanistic and efficacy data in vitro and in animal models, clinical translation remains at an early exploratory stage. As reflected in publicly available registries and recent translational reviews: (*i*) no PDEV-, phytocarrier-, or PBNP-based therapeutic formulation has received marketing authorization from the U.S. FDA, EMA, Pharmaceuticals and Medical Devices Agency (PMDA; in Japan), or other major regulatory agencies; (*ii*) registered human investigations are limited to small, early-phase exploratory trials; and (*iii*) no confirmatory Phase II/III randomized controlled trials demonstrating disease-modifying efficacy have been completed [4,5,6,7,8,9,126,128].

Most registered studies (initiated 2011–2021) have focused on safety and tolerability, feasibility of phytochemical delivery (e.g., curcumin bioavailability), supportive symptom endpoints (e.g., mitigation of oral mucositis), and basic PK parameters. Cohorts have generally ranged from approximately 20 to 60 participants, and long-term outcomes remain unpublished or incompletely reported in the peer-reviewed literature [4,5,6,7,8,11,61].

During the observations from available human data, across early-phase records and registry summaries, the following patterns are consistently reported: acceptable short-term tolerability, no serious adverse events documented in publicly available summaries, no clear signal of acute immunogenicity, no reported dose-limiting toxicities during limited administration periods, and stable hepatic and renal laboratory parameters [8,9,10,11,12,13,14,15,61,126,128].

Primary endpoints have emphasized safety assessment, including adverse event incidence, complement activation markers, cytokine profiling, and clinical chemistry panels, while secondary endpoints have explored delivery feasibility, mucosal barrier effects, or symptom-related measures [8,9,10,11,12,13,14,15,126].

Importantly, in available registry data, no severe infusion reactions, no cumulative toxicity signals, and no documented immune sensitization events have been reported [8,9,10,11,12,13,14,15]. However, interpretation must remain cautious due to small sample sizes, limited follow-up duration, heterogeneous formulations and dosing regimens, and incomplete peer-reviewed reporting for several studies. Accordingly, while preliminary safety signals are encouraging, the current human evidence base is insufficient to establish long-term safety or clinical efficacy.

To delineate the current clinical maturity of plant-derived vesicular systems and distinguish exploratory human data from extensive preclinical literature, Table 10 consolidates verified early-phase trials registered in publicly accessible databases with status information cross-referenced in recent translational reviews [4,5,6,7,8,9,61,126,128]. This structured summary clarifies the limited scale of human investigation and underscores the absence of confirmatory efficacy trials.

Cohorts were small (typically 20–60 participants). Endpoints prioritized safety (adverse events (AEs), biomarkers) over efficacy, with exploratory secondary assessments of delivery or symptom changes. No severe reactions, cumulative toxicities, or sensitization were noted in available records [8,9,10,11,12,13,14,15]. Full peer-reviewed results remain limited or pending for some trials, underscoring the exploratory nature of human data.

##### Critical Limitations of Current Early-Phase Clinical Evidence

While early-phase human studies of PDEVs (Table 10) suggest preliminary tolerability and absence of acute immunogenicity, these findings must be interpreted with caution. Several inherent limitations, typical of first-in-human investigations of novel nanocarrier platforms, constrain their generalizability and confirmatory value:

- Small sample sizes and limited statistical power: trials such as NCT01668849, NCT01294072, and NCT04879810 enrolled modest cohorts (~20–60 participants), limiting the ability to detect rare adverse events, subtle PK variability, or modest efficacy signals. The heterogeneity of patient populations, including head/neck cancer patients undergoing chemoradiation, refractory IBD patients, or individuals receiving colon-targeted curcumin, further restricts generalizability [178,179].
- Safety-focused primary endpoints: these studies primarily assess feasibility and safety (adverse events, cytokine/complement profiles, tissue delivery metrics) rather than disease-modifying efficacy. Secondary endpoints such as symptom scores, inflammation biomarkers, or microbiota changes are exploratory and underpowered, precluding definitive conclusions on clinical benefit [180,181].
- Limited peer-reviewed reporting: many trials remain unpublished in full peer-reviewed form, relying instead on registry summaries or preliminary abstracts. This restricts independent verification, meta-analysis, and robust risk–benefit assessment.
- Exploratory, open-label designs: most studies are single-center, open-label, or non-randomized, introducing potential performance and detection biases. Short follow-up durations limit conclusions on long-term safety, chronic dosing effects, or biodistribution accumulation [11,182].

Table 11 summarizes key early-phase PDEV trials, highlighting interventions, trial status, primary observations, and references. It is intended to provide a clear, at-a-glance view of the current clinical landscape while emphasizing the exploratory nature of these studies.

Taken together, current early-phase evidence is hypothesis-generating rather than confirmatory. No severe safety signals have emerged, supporting PDEVs’ status as immunologically privileged carriers. However, definitive conclusions regarding efficacy, long-term safety, and dose–response relationships remain premature. Advancing PDEVs toward clinical translation requires well-powered, multicenter Phase II/III trials with randomized, placebo-controlled designs, disease-specific primary endpoints (e.g., validated IBD activity indices, mucositis grading scales), and extended follow-up [11,165,178,179,180,181,184].

#### 6.6.2. Safety Considerations and Comparative Profile

Preclinical studies, which dominate the field, and sparse human signals support early favorable characteristics of plant-based carrier systems such as (*i*) immunological compatibility—no detectable anti-PDEV antibodies or hypersensitivity in limited exposures and low complement/T-cell responses; (*ii*) cytotoxicity and genomic safety—high in vitro viability (>90–95%), IC_50_ >500 μg/mL and no evident deoxyribonucleic acid (DNA)/chromosomal damage in available assays; and (*iii*) hemocompatibility and organ-level safety—hemolysis < 5%, stable clinical chemistry parameters, and no short-term histopathological concerns in models [11,12,16,17,18,19,20,22,23].

PK–PD integration, in fact primarily preclinical, with minimal human corroboration, highlights oral uptake via intestinal pathways (e.g., M cells/enterocytes/Peyer’s patches), suggestive improvements in bioavailability and half-life in models, and potential for tissue accumulation and biomarker modulation such as reduced inflammation and microbiota shifts [15,16,17,18,19,20,22,23,24,25].

These features highlight possible advantages for sustainable/oral applications but require significant human validation to substantiate.

#### 6.6.3. Translational Barriers and Mitigation Strategies

Heterogeneity and reproducibility, scalable GMP manufacturing, regulatory classification ambiguity, and mechanistic and human data gaps are the main translational barriers of plant-based carrier systems. Heterogeneity and reproducibility consider the variability from plant source, environment, tissue, and isolation protocols; mitigation strategies include controlled cultivation, elicitation, AI process optimization, and MISEV2023-adapted standards [23]. Low yields and labor-intensive downstream processing influence the recommendation of scalable GMP manufacturing, bioreactor systems and optimized TFF/SEC pipelines targeting higher throughputs as mitigation strategies [24]. Regulatory classification ambiguity refers to hybrid botanical/nanomedicine/biologics identity; in this respect, harmonized chemistry, manufacturing, and controls (CMC) documentation, GLP-compliant toxicology/biodistribution, and early regulatory scientific advice are valuable mitigation strategies [25]. Limited understanding of human uptake, cargo kinetics, off-target effects, and long-term safety is also considered within the framework of mechanistic and human data gaps; mitigation strategies include multi-omics integration, organoid/gut-on-chip models, advanced imaging, and expanded exploratory trials [22,26].

Persistent challenges, such as batch variability, production scalability, regulatory uncertainty, and sparse human evidence, limit near-term clinical impact [3,4,5,7,8,9,34]. Emerging strategies include AI/ML for consistency, clustered regularly interspaced short palindromic repeats (CRISPR)-enabled biogenesis control, metabolic engineering for yields, ISEV-guided standardization, and automated GMP workflows [8,14,17,22,32].

Looking ahead, rigorous larger-scale human studies, mechanistic clarification, and regulatory alignment could enable these platforms to mature into sustainable therapeutics by the early 2030s, targeting inflammation, cancer support, neurodegeneration, and metabolic disorders through a green nanomedicine framework [3,4,5,7,8,9,34].

## 7. Scalability, Green Production, and Regulatory Considerations

Scalability, sustainable manufacturing, and regulatory compliance are critical enablers and persistent bottlenecks for advancing PDEVs, phytocarriers (phytosomes, NLCs/SLNPs, nanoemulsions, mesoporous silica hybrids, green metal/metal oxide NPs) and bioinspired PBNPs from laboratory prototypes to clinically and commercially viable therapeutics. These nature-derived platforms inherently offer renewable biomass feedstocks, low immunogenicity, edibility, multifunctional bioactivity, and favorable safety profiles, distinguishing them from many synthetic nanocarriers and mammalian EVs. Translating these advantages into robust, reproducible industrial processes and regulatory-approved products, however, requires rigorous optimization of bioprocessing, quality standards, environmental footprint, and alignment with evolving regulatory frameworks [8,9,102,185,186,187,188,189,190,191,192,193,194,195].

Despite compelling preclinical evidence and nascent clinical exploration, e.g., early-phase studies using grapefruit, ginger, turmeric, and ginseng-derived carriers for oncology, inflammatory, and metabolic indications, no PDEVs, phytocarriers, or PBNPs have yet achieved full international regulatory approval as a therapeutic. This gap highlights the urgency of addressing scalability, reproducibility, and regulatory acceptance to realize the translational potential of these platforms [12,15,121].

### 7.1. Scalability Challenges and Production Strategies

A primary barrier to clinical translation is scalability and batch consistency. Isolation of vesicles or nanocarriers from field-grown plants is constrained by seasonal and environmental variability (light, soil composition, temperature), inherent biological heterogeneity, and low throughput, which are conditions incompatible with GMP standards [8,10,89]. Large-scale extraction from harvested biomass often yields variable cargo profiles and insufficient quality attributes for pharmaceutical development.

Controlled cellular cultivation systems, such as plant cell suspension cultures, callus cultures, and temporary immersion systems, are increasingly investigated as scalable biomass sources. While detailed bioreactor cultivation parameters remain sparse in the plant EV literature, broader plant biotechnology and nanocarrier studies indicate that in vitro cultivation enhances reproducibility and reduces environmental variability relative to field harvesting [22,26,32,97].

Downstream processing technologies, including TFF, automated SEC, and optimized ultrafiltration, have matured within EV research, supporting higher throughput, enhanced process control, and consistent product profiles at clinical and commercial scales [24,26,31]. Integration of advanced purification workflows reduces operator dependence, limits co-isolates, and reinforces batch-to-batch reproducibility.

#### Production Approaches and Biotechnological Innovations

Despite the growing interest in PDEVs as natural nanocarriers for therapeutic delivery, scalable and reproducible production remains a key translational challenge. Conventional isolation strategies based on freshly harvested plant tissues or juice homogenates typically yield approximately 10^10^–10^13^ vesicles per gram of fresh biomass, depending on plant species, extraction protocol, and purification methodology [33,49,74]. However, downstream isolation workflows, including DUC, DGUC, SEC, or TFF, often result in substantial particle losses (30–70%), limiting overall productivity [42,77,117]. This underscores the need for optimized upstream cultivation systems and scalable purification pipelines capable of ensuring reproducible vesicle yields and quality.

To address variability from field-harvested biomass and seasonal availability, controlled plant cell suspension cultures and liquid cultivation systems have emerged as promising alternatives. In these systems, EVs are secreted directly into the culture medium, enabling non-destructive, repeated harvesting, improved batch-to-batch reproducibility, and continuous production under controlled environmental conditions [53,105]. Moreover, suspension cultures allow precise regulation of nutrients, oxygen, and metabolic activity, supporting stable vesicle secretion during prolonged cultivation. Studies show that plant cell suspension cultures can produce EVs with physicochemical properties comparable to vesicles isolated from whole plant tissues [101,196].

Among advanced biotechnological platforms, temporary immersion bioreactors (TIBs) are particularly promising. Operating through programmed immersion–aeration cycles, these systems allow efficient nutrient uptake while preventing hyper-hydricity and stress. TIBs offer enhanced environmental control, reduced contamination risk, and sustained plant viability, enabling repeated, non-destructive vesicle harvesting [61,108]. Pilot studies involving medicinal plants such as *Pinellia ternata*, *Z. officinale*, and *Glycyrrhiza* spp. demonstrate improved production consistency and energy efficiency compared with conventional plant cultures [101,196].

Elicitation strategies are also explored to enhance vesicle secretion. Controlled abiotic stresses, including salinity, oxidative stress, ultraviolet (UV) irradiation, or hypoxia-mimicking conditions, as well as chemical elicitors, can activate plant defense pathways and stimulate vesicle release [96,123]. Such approaches, widely used to increase secondary metabolite biosynthesis, may lead to 2- to 10-fold increases in vesicle secretion, although robust quantitative datasets for PDEVs remain scarce. Integrating elicitation within controlled bioreactors represents a promising strategy for increased productivity while maintaining vesicle integrity and cargo stability [197,198].

From a bioprocess engineering perspective, hydrodynamic bioreactors designed for mammalian stem cell spheroids have demonstrated high-yield EV production under controlled shear stress and nutrient transport conditions, enabling consistent cargo profiles [199]. While primarily applied to mammalian systems, these principles provide valuable insights into PDEV production, guiding upstream and downstream integration for GMP-compliant manufacturing [101,198].

Despite progress, several challenges remain: (*i*) downstream purification bottlenecks and vesicle heterogeneity, (*ii*) co-isolation of plant macromolecules (polysaccharides, proteins), (*iii*) limited standardization of analytical protocols, and (*iv*) long-term stability of production and reproducibility of vesicle cargo across batches [71,117,136].

Collectively, although industrial-scale PDEV production remains in its early stages, integration of optimized plant culture systems, bioreactor technologies, and efficient purification workflows provides a realistic pathway toward sustainable and clinically relevant manufacturing platforms.

Efficient isolation and large-scale production of PDEVs are critical to translating their therapeutic potential into clinical and industrial applications. Table 12 summarizes current methodologies, highlighting their scalability, technical complexity, and suitability for specific downstream applications. This overview helps researchers select optimal strategies based on intended use, resource availability, and desired cargo fidelity.

Scalable, reproducible, and cargo-preserving isolation methods are essential to unlock the therapeutic potential of PDEVs, and recent advances in bioreactor design and physicochemical modulation provide promising paths for industrial translation [101,196,197,199].

### 7.2. Green Production and Sustainability Principles

Sustainability is intrinsic to plant-based carrier platforms and aligns with green chemistry and circular economy principles. Green manufacturing emphasizes aqueous or solvent-minimized extraction, enzymatic or supercritical fluid processing, and the use of renewable feedstocks to reduce environmental impact. For example, green synthesis approaches that utilize plant polyphenols or other natural reducing agents for metallic NPs formation (e.g., Au, Ag, Fe_3_O_4_) are increasingly adopted due to their minimal reliance on hazardous reagents [102,104].

Bioreactor-based production further enhances sustainability by reducing land use, water consumption, and agricultural inputs compared with conventional cultivation. Although life cycle assessment (LCA) data specific to plant EV production is limited, analogous studies suggest that controlled bioreactor cultivation and aqueous extraction can substantially lower the carbon footprint relative to traditional nanocarrier platforms [32,97,102].

Waste valorization, including the use of fruit pomace, peels, spent herbs, and other agro-industrial residues as feedstocks for EV or PBNP extraction, exemplifies circularity and can reduce material costs while generating additional value from biomass by-products [9,102].

### 7.3. Regulatory Considerations and Quality Standardization

Regulatory pathways for plant-derived nanocarriers remain case-specific due to their complex, hybrid nature. U.S. FDA evaluates such products under existing frameworks for nanomaterials and botanical drugs, requiring comprehensive characterization of physicochemical properties, GLP-compliant toxicology and safety data, biodistribution profiles, and stringent manufacturing controls. Similarly, EMA assesses complex nanomedicines under nanotechnology and biologics guidance, emphasizing GMP compliance, risk-based quality systems, and environmental safety considerations [128].

Community-driven frameworks, such as the MISEV2023, provide standardized guidance on isolation methods, particle characterization (size, concentration, surface markers), and functional assays. While not exclusively developed for plant systems, MISEV guidelines offer a structured baseline increasingly adopted by plant EV researchers, enhancing cross-study comparability and supporting regulatory engagement [15,74,123].

Ongoing efforts within the EV community advocate for plant-specific reference materials, controls, and nomenclature to address unique challenges, including cell wall contaminants, matrix co-isolates, and cargo heterogeneity inherent to botanical sources [74,123].

### 7.4. Outlook and Pathways Toward Regulatory Maturity

Looking toward 2030, integrated manufacturing ecosystems combining controlled bioprocessing, advanced purification, predictive analytics, and standardized quality frameworks are expected to reduce barriers to clinical translation and regulatory approval. Harmonization of EV characterization standards with regulatory expectations will accelerate learning across sectors, enabling comparative datasets that underpin safety, efficacy, and consistency claims [97,128].

As plant-based carriers enter higher-phase clinical trials, accumulation of robust toxicology, biodistribution, and PK data will further clarify regulatory pathways and manufacturing expectations. Supported by community standards and proactive regulatory engagement, PDEVs, phytocarriers, and PBNPs are positioned as promising contributors to the next generation of nanomedicine, particularly for oncology, inflammatory, neurodegenerative, and metabolic disorders [12,15,128,185,186,187,188,189,190,191,192,193,194,195].

#### 7.4.1. Risks and Foundational Considerations for Emerging Optimization Technologies

Precision engineering of PDEVs requires the convergence of targeted genetic interventions and advanced process modeling. CRISPR genome editing allows direct control of vesicle biogenesis and cargo composition, while AI-driven optimization ensures reproducible, high-yield, and scalable production. Together, these strategies offer a robust framework for translating PDEV-based therapeutics from laboratory research to industrial and clinical applications.

CRISPR-mediated genome editing in plant systems offers substantial potential for rational engineering of PDEVs. By precisely targeting key biogenesis pathways, including multivesicular body trafficking and vesicle secretion machinery, CRISPR strategies can enhance vesicle yield, modulate cargo composition (e.g., therapeutic miRNAs, secondary metabolites), and reduce vesicle heterogeneity through targeted genetic modifications. Pilot studies in model plant lines have demonstrated that CRISPR-based interventions can improve production of bioactive metabolites or stress-responsive vesicles, establishing feasibility for translational PDEV optimization [88,200,201,202].

Despite its promise, CRISPR/CRISPR-associated protein (Cas) technology carries significant risks, most notably off-target effects—unintended edits at non-homologous genomic sites. Such events can lead to unexpected phenotypic alterations, impaired plant physiology, or accumulation of deleterious mutations across generations [200,203]. Off-target frequencies depend on guide RNA design, choice of Cas variants (e.g., high-fidelity SpCas9-HF1, base editors, or prime editors, which reduce but do not eliminate risk), delivery strategy, and genome complexity [201,204,205]. Additional considerations include regulatory classification (e.g., genetically modified organism (GMO) status), epigenetic stability, and potential long-term ecological consequences if edited lines are deployed at scale [2,201,204].

Mitigation requires comprehensive validation, including whole-genome sequencing, off-target detection via GUIDE-seq or CIRCLE-seq, and multi-generational phenotyping to ensure phenotypic stability and biosafety for therapeutic-grade PDEVs [202,204,206]. Complementary strategies, such as vesicle-mediated delivery of CRISPR components, enhance targeting precision while minimizing cytotoxicity [205,206]. Minimizing off-target edits remains a key research priority for translating CRISPR-based engineering into safe, reproducible PDEV production.

AI-driven process optimization provides a complementary approach for improving PDEV manufacturing. AI models are constructed using high-quality, standardized datasets that integrate historical process parameters and functional readouts. Essential input data include physicochemical measurements (e.g., particle size and concentration via NTA), multi-omics profiles (proteomics, lipidomics, small RNA-seq), and functional cargo bioactivity. Transfer learning from mammalian EVs or synthetic data augmentation can improve model generalization for plant-derived systems [4,5,207,208].

Hybrid AI models combining mechanistic and data-driven approaches are increasingly favored. Mechanistic modeling, such as kinetic or mass-balance equations describing vesicle biogenesis, is integrated with ML methods, including reinforcement learning for dynamic control of bioreactor parameters (pH, dissolved oxygen, shear stress, elicitation timing, and medium composition) and deep learning (DL) models, e.g., convolutional neural networks, for inline spectroscopy monitoring of vesicle heterogeneity. These approaches have demonstrated >90% yield consistency and <5% batch-to-batch variability in mammalian and microbial EV systems, supporting translation to plant-derived vesicles [3,4,207,208].

Current limitations include sparse plant-specific datasets, interpretability of black-box models, and validation of scalability. Hybrid mechanistic-data-driven modeling not only improves predictability but also aligns with regulatory expectations by enabling explainable and auditable process decisions, which is critical for GMP-compliant PDEVs manufacturing [205,207,208].

Integrating CRISPR genome editing with AI-driven process optimization enables precise, reproducible, and scalable engineering of PDEVs [200,201,202]. CRISPR allows genotype-specific modulation of vesicle biogenesis and cargo composition, including therapeutic small RNAs and bioactive metabolites, while AI models provide predictive, real-time control of complex bioprocess parameters, such as pH, dissolved oxygen, shear stress, and elicitation timing [206,207,208]. Together, these complementary strategies overcome major bottlenecks (low yield, heterogeneity, and process unpredictability), facilitating the production of safe, therapeutically effective, and GMP-compliant PDEVs [7,9,204]. Table 13 summarizes the key objectives, potential risks, and mitigation approaches for both CRISPR and AI strategies in PDEVs optimization, providing a concise overview of current considerations and best practices.

#### 7.4.2. Clinical Translation, Intellectual Property, and Commercialization Prospects of Plant-Derived Nanocarriers

Future research on plant-derived nanocarriers must extend beyond methodological optimization to strategically address clinical translation, intellectual property (IP), and commercialization pathways. These dimensions are critical to convert the scientific potential of PDEVs into clinically and commercially viable therapeutics.

Despite robust preclinical evidence supporting efficacy in inflammation, oncology, neurological disorders, and regenerative medicine [11,15,136,151], clinical data remain sparse. Current human trials, such as grape-derived vesicles for oral mucositis and ginger/turmeric vesicles for IBD, demonstrate excellent safety, tolerability, and preliminary PK feasibility [7,167]. To accelerate regulatory progression, future studies should prioritize (*i*) Phase II/III randomized trials powered for efficacy with disease-relevant endpoints, e.g., IBD activity indices, response evaluation criteria in solid tumors (RECIST), or CNS functional outcomes [7,128]; (*ii*) biomarker-guided stratification using PDEV cargo signatures, including miRNA-based responder prediction [9,14]; (*iii*) early regulatory engagement to define product classification, critical quality attributes (CQAs), and potency assays aligned with therapeutic claims [74,97,123]; and (*iv*) adaptive trial designs incorporating real-world evidence and accelerated pathways for high-unmet-need indications [129].

As PDEVs transition from discovery to industrial development, strategic IP management becomes crucial. Recent patent activity spans isolation techniques, engineered hybrids, cargo-loading technologies, and therapeutic applications [5,133,140]. Key strategies include (*i*) platform-level protection for core technologies (standardized isolation pipelines, hybrid vesicles) while filing narrower, indication-specific claims; (*ii*) defensive publishing of non-core innovations to maintain freedom-to-operate [168]; (*iii*) global IP coverage via Patent Cooperation Treaty (PCT) filings targeting major markets with distinct regulatory/reimbursement landscapes [31,149]; and (*iv*) collaborative commercialization models such as licensing or co-development partnerships with industry [140,141].

PDEVs benefit from renewable sourcing, scalable production compared to mammalian EVs, and precedents in phytosome commercialization [37,38,45]. Market projections indicate strong growth for exosome/nanovesicle therapeutics and functional foods [135,186]. Critical challenges include (*i*) GMP-compliant scale-up and cost-effective manufacturing [31,32,152]; (*ii*) pharmacoeconomic evidence to support reimbursement [97,138]; and (*iii*) differentiation within competitive nanocarrier landscapes, including synthetic NPs, phytosomes, and plant-derived metallic NPs [41,48,65].

Successful commercialization will likely require public–private partnerships, spin-offs focusing on flagship indications (e.g., IBD or mucositis), and hybrid business models combining rapid nutraceutical entry with phased prescription drug development. Early investment in robust CMC documentation, scalable bioreactors, and real-world evidence generation will be decisive.

Future programs should align technical, translational, and commercial dimensions to ensure impact as follows: (*i*) harmonized pipelines spanning formulation, preclinical validation, regulatory readiness, and clinical translation [139,189,191]; (*ii*) robust CMC frameworks, stability profiling, and quality control strategies consistent with MISEV2023 guidelines [123,124,137]; and (*iii*) interdisciplinary commercialization models, including nutraceutical-to-pharmaceutical progression, academic–industry partnerships, and spin-offs targeting high-value therapeutic niches [100,104,169].

In summary, the successful translation of plant-derived nanocarriers into clinically and commercially viable therapeutics demands the synchronized integration of rigorous clinical evaluation, proactive IP strategies, and scalable, GMP-compliant manufacturing, alongside ongoing technical and mechanistic innovations. This multidimensional approach ensures that PDEVs can fulfill their promise as sustainable, precision phytotherapeutics with reproducible efficacy and safety profiles.

Ultimately, the convergence of scalable production, green manufacturing, and defined regulatory frameworks is essential to realize plant-derived nanocarriers as sustainable, safe, and effective modalities in precision therapeutics [186,187,188,189,190,191,192,193,194].

## 8. Conclusions

Plant-based carrier systems, encompassing PDEVs, engineered phytocarriers, and bioinspired polymeric PBNPs, represent a transformative advance in sustainable nanomedicine. By uniting intrinsic botanical bioactivity with the precision, tunability, and versatility of modern nanotechnology, these platforms deliver exceptional biocompatibility, GI stability, targeted delivery, and cross-kingdom therapeutic effects. Relative to conventional synthetic carriers, they provide superior oral bioavailability, prolonged circulation, disease-site accumulation, and synergistic action, all with outstanding safety. Recent preclinical and early-phase clinical studies highlight their broad potential in oncology, inflammatory, metabolic/hepatic, neurodegenerative, and regenerative applications, with hybrid/stimuli-responsive designs enabling magneto-targeting, controlled release, and combination therapies. Looking ahead, promising advances in CRISPR-based plant engineering for yield and cargo optimization, AI-driven personalization and process control, continuous bioreactor production, and harmonized regulatory frameworks will accelerate translation. These systems exemplify a paradigm aligning pharmaceutical innovation with environmental sustainability, harnessing renewable resources for scalable, low-cost, immunologically privileged nanotherapeutics. In summary, plant-based carriers bridge centuries of pharmacognostic wisdom with cutting-edge nanotechnology, heralding a green, equitable era of precision medicine with improved outcomes, wider access, and a more sustainable drug delivery future.

## Figures and Tables

**Table 1:** Green synthesized metal/metal oxide NPs: short-term safety findings vs. long-term data gaps.

Nanoparticle Type	Plant Capping/Source	Reported Short-Term Findings	Chronic/Long-Term Data	Immunogenicity Data	References
AgNPs	various plant extracts	high viability; low acute ROS/cytokine induction	scarce	minimal/not assessed	[79,81,86]
AuNPs	green tea, medicinal herbs	low cytotoxicity in vitro; acute tolerance	none reported	limited	[82,85]
ZnO NPs	neem extract, others	antimicrobial; tolerated in short in vivo models	only acute/subacute	not evaluated	[82,87]
Fe_3_O_4_ NPs	spinach, plant extracts	biocompatible MRI contrast; limited acute toxicity	sparse data	limited	[80,84]

AgNPs: Silver nanoparticles; AuNPs: Gold nanoparticles; Fe_3_O_4_: Magnetite; MRI: Magnetic resonance imaging; NPs: Nanoparticles; ROS: Reactive oxygen species; ZnO: Zinc oxide.

**Table 2:** Mechanistic differentiation between intrinsic bioactive components and carrier function in PDEVs.

Mechanistic Category	Representative Components	Functional Contribution	Representative Systems and Effects	References
endogenous small RNAs (miRNAs)	plant miRNAs	cross-kingdom regulation of inflammatory and metabolic gene expression	PDEVs uptake modulates target gene networks in mammalian cells	[9,11,12,13,14]
bioactive lipids	PA, PC, PE	influences membrane fusion, uptake pathways; potential immune modulation	PA-rich PDEVs preferentially internalized by macrophages	[10,11,95]
secondary metabolites	gingerols, polyphenolics	direct antioxidant and anti-inflammatory activity	enriched metabolites in PDEVs linked to ROS reduction and immunomodulation	[5,90,92,96]
proteins	vesicle transporters, chaperones	structural stabilization, potential uptake facilitation	proteomic signatures associated with vesicle integrity and interaction with recipient cells	[7,16,91]
vesicle structure (carrier function)	lipid bilayer nanovesicle membrane	encapsulation/protection, enhanced internalization, biodistribution	improved stability and systemic delivery of intrinsic cargo/extrinsic therapeutics	[5,8,11,94]

Intrinsic cargo refers to naturally encapsulated molecules within PDEVs; carrier function refers to delivery properties derived from vesicle physical structure. Representative effects include biological pathways influenced by intrinsic cargo vs. pharmacokinetic or cellular uptake properties of vesicles. miRNAs: Micro-ribonucleic acids; PA: Phosphatidic acid; PC: Phosphatidylcholine; PDEVs: Plant-derived extracellular vesicles; PE: Phosphatidylethanolamine; RNAs: Ribonucleic acids; ROS: Reactive oxygen species.

**Table 3:** Botanical sources and species-specific mapping: medicinal plants, main phytochemicals, associated nanocarrier platforms, intrinsic/carrier-enhanced bioactivities, and potential translational applications.

Family	Species	Main Phytochemicals	Carrier Platforms (Condensed)	Intrinsic/Carrier-Enhanced Bioactivity	Translational Application	References
*Zingiberaceae*	*Zingiber officinale*	gingerols, shogaols	PDEVs, nanoemulsions, NLCs, hybrid systems	anti-inflammatory, mucosal protective; enhanced GI stability and epithelial uptake	IBD, inflammatory disorders	[47,58,142]
	*Curcuma longa*	curcuminoids	phytosomes, NLCs, SLNPs, mesoporous silica hybrids, green AgNPs/AuNPs	NF-κB inhibition, antioxidant, proapoptotic; 5–20-fold higher bioavailability, BBB penetration	cancer, arthritis, neurodegeneration	[5,8,13,33,60,65,90,143]
*Araliaceae*	*Panax ginseng*	ginsenosides	PDEVs, phytosomes, lipid nanocarriers	immunomodulatory, metabolic regulation; improved systemic exposure	fatigue, metabolic syndrome	[4,38]
*Alliaceae*	*Allium sativum*	allicin, organosulfur compounds	nanoemulsions, SLNPs, green metallic NPs	broad-spectrum antimicrobial; improved biofilm penetration	resistant infections	[55]
*Rutaceae*	*Citrus* × *paradisi*	flavonoids, limonoids	PDEVs, biomimetic EVs drug carriers	antioxidant, vascular protective; targeted delivery	cardiometabolic disease	[144,145]
*Hypericaceae*	*Hypericum* *perforatum*	hypericin, hyperforin, flavonoids	PDEVs, SLNPs, NLCs, nanoemulsions, liposomes, polymeric NPs, green metallic NPs	antidepressant, anti-inflammatory, photodynamic cytotoxicity; enhanced neuronal uptake and ROS-mediated PDT efficacy	depression, neuroinflammation, photodynamic oncology, WH, resistant infections	[106,107,108,109,110]
*Nelumbonaceae*	*Nelumbo nucifera*	flavonoids, alkaloids	lotus-derived EVs	anti-inflammatory, WH modulation; enhanced tissue regeneration	WH, inflammatory disorders	[111]
*Rosaceae*	*Malus domestica*	polyphenols	apple-derived EVs	antioxidant, immunomodulatory; improved EVs characterization	inflammation, metabolic disorders	[99]
*Theaceae*	*Camellia sinensis*	EGCG, catechins	PDEVs, phytosomes, green AuNPs	antioxidant, anti-proliferative; prolonged half-life, enhanced uptake	cancer, osteoporosis	[60,89]
*Solanaceae*	*Withania somnifera*	withanolides	phytosomes, zein/chitosan PBNPs	adaptogenic, neuroprotective; BBB-compatible sustained release	stress, neuroinflammation	[40,61,66,67]
*Ginkgoaceae*	*Ginkgo biloba*	ginkgolides, bilobalides	phytosomes, mesoporous silica hybrids, PDEVs	antiplatelet, mitochondrial stabilization; enhanced CNS delivery	Alzheimer’s disease	[5,143]
*Asteraceae*	*Silybum marianum*	silymarin	phytosomes	hepatoprotective, antifibrotic; enhanced membrane permeability and lymphatic uptake	NAFLD, fibrosis	[10,90]
	*Echinacea purpurea*	alkylamides	PDEVs, mesoporous silica hybrids	cytokine modulation; targeted mucosal delivery	allergy, autoimmunity	[34,48,136]
*Meliaceae*	*Azadirachta indica*	azadirachtin	green synthesized metal NPs	antiparasitic, anticancer; siRNA co-delivery, improved intracellular targeting	malaria, oncology	[7,33,42,54]
*Fabaceae*	*Lupinus luteus*	phenolics, defense metabolites	AuNPs	antifungal defense activation; AuNPs-mediated enhancement of host resistance	agricultural biotechnology, antifungal strategies	[112]
*Lamiaceae*	*Sideritis scardica*	flavonoids	clinoptilolite hybrid systems	antioxidant, neuroprotective; zeolite-mediated sustained release	neurodegeneration	[3,4,5,58]
*Portulacaceae*	*Portulaca oleracea*	polyphenols	Fe_3_O_4_-based hybrids	antioxidant, cytotoxic; magnetic targeting, ROS-amplified cytotoxicity	targeted anticancer	[3,4,5,59]
*Ranunculaceae*	*Helleborus* *purpurascens*	steroidal glycosides, polyphenols	chitosan-based carriers	antioxidant, cytotoxic; mucoadhesive, enhanced uptake	anticancer, oxidative stress modulation	[113]
*Brassicaceae*	*Armoracia rusticana*	glucosinolates, isothiocyanates	kaolinite-based phytocarriers	antitumoral, antioxidant; controlled adsorption-release, cytotoxic selectivity	oncology	[114]
*Apiaceae*	*Heracleum* *sphondylium*	furanocoumarins, polyphenols	AgNPs-based carriers	antioxidant, antimicrobial, cytotoxic; ROS modulation, increased tumor susceptibility	antimicrobial, anticancer	[115]
*Santalaceae*	*Viscum album*	lectins, viscotoxins, phenolics	AuNPs-engineered carriers	immunomodulatory, cytotoxic; AuNPs stabilization, enhanced intracellular delivery	oncology, immunotherapy adjunct	[116]
*Papaveraceae*	*Chelidonium majus*	isoquinoline alkaloids, flavonoids	AuNPs-based carriers	antimicrobial, cytotoxic; AuNPs-mediated stabilization, enhanced cellular interaction	antimicrobial therapy, oncology	[117]

AgNPs: Silver nanoparticles; AuNPs: Gold nanoparticles; BBB: Blood–brain barrier; CNS: Central nervous system; EGCG: Epigallocatechin-3-gallate; EVs: Extracellular vesicles; Fe_3_O_4_: Magnetite; GI: Gastrointestinal; IBD: Inflammatory bowel disease; NAFLD: Non-alcoholic fatty liver disease; NF-κB: Nuclear factor-kappaB; NLCs: Nanostructured lipid carriers; NPs: Nanoparticles; PBNPs: Plant-based nanoparticles; PDEVs: Plant-derived extracellular vesicles; PDT: Photodynamic therapy; ROS: Reactive oxygen species; siRNA: Small interfering ribonucleic acid; SLNPs: Solid lipid nanoparticles; WH: Wound healing.

**Table 4:** Comparative assessment of major plant-derived nanocarrier classes: translational attributes, including scalability, cost, therapeutic performance, safety/biocompatibility, and clinical/regulatory maturity (qualitative scoring based on 2023–2026 literature).

Carrier Type	Scalability (Production Yield and Reproducibility)	Cost (Manufacturing and Sourcing)	Efficacy (Bioavailability, Bioactivity, and Targeting)	Safety/ Biocompatibility	Translational Readiness (Clinical Evidence and Regulatory Pathway)	Key Strengths and Limitations	References
PDEVs	3 (moderate; batch variability; improving with bioreactors/TIBs; low yields ~10^10^–10^13^ vesicles/g tissue)	4 (high; renewable/edible sources, low reagent cost; labor-intensive isolation)	5 (excellent; intrinsic cross-kingdom activity, high encapsulation versatility, GI stability, BBB potential)	5 (excellent; GRAS/edible, low immunogenicity, negligible toxicity)	3 (moderate; early-phase trials only; standardization gaps)	strengths: dual therapeutic/carrier role, sustainability; limitations: heterogeneity, purification challenges	[7,8,9,10,11,15,26,27,28,29,30,31,32,74,75,76,146,147]
phytosomes/phospholipid complexes	5 (high; stoichiometric complexation, scalable, GMP-like methods)	5 (high; low-cost phospholipids; commercial examples like Meriva^®^)	4 (good; 5–20-fold higher bioavailability via lymphatic uptake, enhanced stability; strong in inflammation/hepatoprotection)	4 (good; biocompatible, clinical safety data; some enzymatic resistance concerns)	5 (high; multiple clinical products, proven pharmacokinetics)	strengths: mature technology, clinical evidence; limitations: less intrinsic multifunctionality than PDEVs	[37,38,39,40,41,42,43,44,45,90,101]
SLNPs	4 (good; scalable via high-pressure homogenization; some polymorphism)	4 (good; affordable lipid matrices; established processes)	4 (good; sustained release, high encapsulation ~80%; improved solubilization/stability)	4 (good; biocompatible; low toxicity, potential gelation)	4 (good; preclinical/clinical in various fields; easier regulatory path than hybrids)	strengths: controlled release, stability; limitations: lower drug loading vs. NLCs	[5,46,48,49,50]
NLCs	5 (high; imperfect matrix avoids expulsion; scalable like SLNPs, superior)	4 (good; similar to SLNPs; minor cost from liquid lipids)	5 (excellent; higher loading ~90%, better release >90%)	4 (good; biodegradable; enhanced stability reduces toxicity risks)	4 (good; growing preclinical data; strong oral/ topical delivery)	strengths: overcomes SLNPs limitations, high performance; limitations: slightly complex formulation	[5,35,46,48,49,50]
green synthesized metal/metal oxide NPs (Ag, Au, ZnO, Fe_3_O_4_)	4 (good; one-pot plant extract reduction; scalable but purity/ion release variability)	5 (high; low-cost plant reductants; no harsh chemicals)	4 (good; synergistic bioactivity, magnetic targeting, antimicrobial/cytotoxic)	3 (moderate; good corona mitigation but potential long-term cytotoxicity/ROS concerns)	3 (moderate; preclinical synergy strong; regulatory hurdles)	strengths: dual functionality, low-cost green synthesis; limitations: safety data gaps, less oral suitability	[48,49,50,51,52,53,54,59]
bioinspired polymeric PBNPs (e.g., zein, chitosan)	4 (good; self-assembly/scalable; mucoadhesive, optimization needed)	4 (good; abundant plant polymers; biodegradable)	4 (good; 80–95% encapsulation, sustained release; colon-targeting)	4 (good; biodegradable, mucoadhesive; high viability)	3 (moderate; strong preclinical; limited clinical data)	strengths: tunable, stimuli-responsive; limitations: long-term safety underexplored	[65,66,67,68,69,70,71,72,73]
hybrid phyto- inorganic composites (plant extract–silica/ –clinoptilolite/ –metal)	3 (moderate; multi-step integration; tunable but complex scaling)	3 (moderate; low-cost components but added processing)	5 (excellent; emergent synergy, controlled release, multifunctionality)	3 (moderate; enhanced by phytochemical corona; inorganic risks persist)	2 (limited; mostly preclinical; regulatory complexity)	strengths: theranostic potential; limitations: mechanistic gaps, scalability challenges	[13,47,58,59,60,113,114,115,116,117,142]

Scores (1–5) represent relative positioning based on the current literature (2023–2026), highlighting strengths and developmental constraints. Scoring scale: 1—low; 2—limited; 3—moderate; 4—good; 5—high/advanced. Ag: Silver; Au: Gold; BBB: Blood–brain barrier; Fe_3_O_4_: Magnetite; GI: Gastrointestinal; GMP: Good Manufacturing Practices; GRAS: Generally recognized as safe; NLCs: Nanostructured lipid carriers; NPs: Nanoparticles; PBNPs: Plant-based nanoparticles; PDEVs: Plant-derived extracellular vesicles; ROS: Reactive oxygen species; SLNPs: Solid lipid nanoparticles; TIBs: Temporary immersion bioreactors; ZnO: Zinc oxide.

**Table 5:** Comparative translational attributes of major plant-derived nanocarrier platforms re-ported in the recent literature.

Nanocarrier Platform	Structural Characteristics	Typical Cargo	Key Advantages	Reported Limitations	Representative Evaluation Models	References
PDEVs	natural lipid bilayer vesicles containing plant lipids, proteins and RNA	phytochemicals, nucleic acids	high biocompatibility, GI stability, intrinsic biological activity	isolation standardization challenges; variability between plant sources	Caco-2 epithelial cells, RAW264.7 macrophages, murine oral delivery models	[8,9,10,11]
phytosomes	phytochemical–phospholipid molecular complexes	polyphenols, flavonoids	improved membrane permeability and oral bioavailability	limited intrinsic targeting	rodent PK studies, human PK, intestinal permeability assays	[37,38]
SLNs	NPs composed of solid lipid matrices stabilized by surfactants	lipophilic phytochemicals	high stability and protection from degradation	limited loading capacity	release studies, permeability assays, animal PK models	[92,95]
NLCs	hybrid lipid systems with solid and liquid lipid phases	lipophilic phytochemicals	higher loading capacity and improved release profiles	formulation complexity	cellular uptake assays, PK models	[92,95]
polymeric plant-based NPs	biopolymer-based carriers (zein, chitosan)	polyphenols, nutraceuticals	mucoadhesion and colon-targeting potential	limited clinical translation	Caco-2 transport models	[94]
self-assembled phytochemical nanostructures	nanostructures formed by spontaneous molecular assembly	flavonoids, alkaloids	high loading efficiency, minimal excipients	stability challenges	cellular uptake and solubility studies	[93]

GI: Gastrointestinal; NLCs: Nanostructured lipid carriers; NPs: Nanoparticles; PDEVs: Plant-derived extracellular vesicles; PK: Pharmacokinetic; RNA: Ribonucleic acid; SLNPs: Solid lipid nanoparticles.

**Table 6:** Proposed standardized framework for comparative evaluation of plant-derived nanocarriers.

Evaluation Parameter	Proposed Standardized Model	Analytical Endpoints	Purpose	References
oral bioavailability	rodent PK studies using identical phytochemical cargo (e.g., curcumin)	AUC_0–*t*_, C_max_, *t*_1/2_, relative bioavailability	quantitative comparison of systemic exposure	[91,92]
intestinal permeability	Caco-2 monolayer transport assays	apparent permeability coefficient (Papp)	evaluate absorption potential	[11]
cellular uptake	RAW264.7 macrophages, THP-1 cells, HepG2 hepatocytes	% internalization, fluorescence intensity	compare cellular uptake efficiency	[12,148]
biodistribution	in vivo fluorescence imaging in rodent models	tissue accumulation profiles	evaluate targeting and distribution	[91]
stability in GI environment	simulated gastric and intestinal fluids	particle integrity and release kinetics	assess oral delivery robustness	[92]

AUC_0–*t*_: Area under the curve from time zero to the last measurable concentration (at time *t*); C_max_: Maximum concentration; GI: Gastrointestinal; PK: Pharmacokinetic; *t*_1/2_: Half-life.

**Table 7:** Comparative overview of main isolation and purification techniques of PDEVs.

Method	Principle	Advantages	Limitations	Representative Plant Examples and Applications	Recent Innovations/Improvements	References
DUC	sequential centrifugal sedimentation by size/ density	high yield, large-scale capability, cost-effective	time-consuming (4–6 h), moderate recovery (30–60%), shear- induced damage, co-isolation of proteins/organelles	*Panax ginseng* (immunomodulation), *Echinacea purpurea* (immunostimulation), *Solanum lycopersicum*, *Brassica oleracea* (anti-inflammatory, antitumor)	double ultracentrifugation for roots; integration with microfiltration for viscous extracts	[3,4,5,11,18,19,26,27,28,32,33,34,35,36,37,38,39,40,41,42,43,44,45,47,51,52,53,61,62,63,118,121,123,124,125,138,140,149,150,151,152,153]
DGUC	buoyant density separation in continuous/discontinuous gradients (iodixanol, sucrose, Percoll)	superior purity (>95%), subpopulation separation, recovery up to 60%	low throughput, complex setup, potential bioactivity loss (sucrose)	*Camellia sinensis* (EGCG, antioxidant/anti-osteoporotic), *Matricaria chamomilla* (apigenin, sedative/anti-inflammatory), tomato/citrus (naringin/limonene, leukemia sensitization)	pre-clearing steps; iodixanol gradients for heterogeneous populations; hybrid phytocarrier purification	[3,4,5,11,18,19,26,27,28,32,33,34,35,36,37,38,39,40,41,42,43,44,45,47,51,52,53,61,62,63,118,121,123,124,125,138,140,149,150,151,152,153]
SEC	size-based separation through porous media	gentle, high recovery (>70–90%), rapid (30–60 min), minimal shear damage	limited sample capacity, equipment cost, co-elution of similarly sized contaminants	*Mentha* × *piperita*, *Rosmarinus* *officinalis* (menthol/carnosic acid, analgesic/neuroprotective), *Punica granatum*, *Brassica oleracea* (punicalagins/ sulforaphane, antioxidant/ antitumor)	pellet-free protocols for viscous extracts; hybrid phytocarrier purification (e.g., quercetin–AuNPs)	[3,4,5,11,18,19,22,25,26,27,28,32,33,34,35,36,37,38,39,40,41,42,43,44,45,47,51,52,53,61,62,63,119,121,123,124,125,138,140,141,149,150,151,152,153]
PEG precipitation	polymer-induced vesicle aggregation (6–8 kDa PEG, 8–10%)	cost-effective, simple, scalable	co-precipitation of proteins/nucleic acids, subpopulation bias	*Syzygium aromaticum*, *Cinnamomum verum* (eugenol/cinnamaldehyde, analgesic/antidiabetic), *Solanum nigrum*, *Pachyrhizus erosus* (anti-inflammatory/anti-melanogenic)	combined with filtration (0.22–0.45 μm) for higher purity; hybrid PDEVs carrier assembly	[26,27,28,31]
UF/TFF	size-based membrane filtration (0.1–0.8 μm, 100 kDa cutoff), TFF prevents clogging	rapid, gentle, high recovery, scalable	membrane adsorption, potential shear damage	*Nelumbo nucifera* (nuciferine, anti-obesity), cabbage (anti- inflammatory), hybrid carriers (curcumin–MSNPs, silymarin–Fe_3_O_4_, EGCG–GO)	dcTFF for large-volume fruits; separation of hybrid inorganic– organic carriers	[32,33,34,38,43,44,45]
enzymatic digestion	cell wall breakdown (cellulase, pectinase)	targeted vesicle release, increased yield	plant-specific optimization required; risk of over-digestion	*Azadirachta indica* (azadirachtin, antiparasitic), *Moringa oleifera* (quercetin, antihypertensive), hybrid carriers (berberine– clinoptilolite, apigenin–HNTs)	root tissue-specific enzymatic digestion; combined with hybrid carrier assembly	[40,41,51,52,53]
UAE	cavitation enhances vesicle release	boosts yield and stability	energy-intensive, risk of overheating	*Glycine max*, *Nelumbo nucifera*, *Serenoa repens*, *Silybum* *marianum*, hybrid carriers (quercetin–AuNPs, resveratrol–ZnO)	combined with DUC; enhanced vesicle stability and hybrid carrier preparation	[42,43,44,61,62,63]
IA capture	biomarker- specific Ab binding	high specificity, subclass enrichment	low yield, Ab dependency	*Arabidopsis* (TET8+ vesicles, RNA cargo), *Panax ginseng* (subpopulation isolation)	subpopulation-specific small RNA studies	[46]

Ab: Antibody; AuNPs: Gold nanoparticles; dcTFF: Double-cyclic tangential flow filtration; DGUC: Density gradient ultracentrifugation; DUC: Differential ultracentrifugation; EGCG: Epigallocatechin-3-gallate; Fe_3_O_4_: Magnetite; GO: Graphene oxide; HNTs: Halloysite nanotubes; IA: Immunoaffinity; MSNPs: Mesoporous silica nanoparticles; PDEVs: Plant-derived extracellular vesicles; PEG: Polyethylene glycol; RNA: Ribonucleic acid; SEC: Size exclusion chromatography; TET8: Tetraspanin 8; TFF: Tangential flow filtration; UAE: Ultrasound-assisted extraction; UF: Ultrafiltration; ZnO: Zinc oxide.

**Table 8:** Engineering strategies for PDEVs and hybrid plant-based nanocarriers: methods, typical efficiency ranges, functional enhancements, and applications in preclinical studies.

Strategy	Method	Typical Efficiency/ Enhancement	Functional Outcome	Applications	References
cargo loading	passive incubation	30–70% encapsulation	delivery efficiency (green)	small molecules, siRNA, miRNA	[75,133]
	electroporation	60–90% loading	enhanced nucleic acid delivery (green)	chemotherapeutics, RNA therapeutics	[75,133]
	sonication	50–80% loading	improved hydrophobic drug incorporation (green)	hydrophobic small molecules	[75,154]
surface functionalization	peptide (cRGD) conjugation	1.5–3-fold higher tumor uptake	targeted delivery (green)	cancer therapy	[133,154]
	aptamer modification	1.5–3-fold higher receptor-specific binding	targeted uptake (green)	RNA/siRNA delivery	[75,133]
hybrid engineering	lipid fusion/PEGylation	extended circulation: 1.5–2-fold higher half-life	stability/sustained release (blue)	controlled release therapeutics	[75,133]
	inorganic–organic hybrids	multifunctional: drug and imaging	therapy and diagnostic capability (orange)	imaging-guided therapy, neuroprotection, antioxidant delivery	[58,59,60,113,114,115,116,117,142,154]

cRGD: Cyclic arginine-glycine-aspartate; miRNA: Micro-ribonucleic acid; PDEVs: Plant-derived extracellular vesicles; PEG: Polyethylene glycol; RNA: Ribonucleic acid; siRNA: Small interfering ribonucleic acid.

**Table 9:** Summary of stability, biocompatibility, cytotoxicity, and genotoxicity profiles of representative plant-based carrier systems.

Assay Type	Key Metrics	Representative Plants/Carriers	Outcomes	References
stability (DLS/NTA post- incubation)	size retention > 90%, PDI < 0.3	ginger, grapefruit, turmeric NLCs; *Portulaca*–Fe_3_O_4_ hybrid; *Heracleum*-, *Chelidonium*-, *Viscum*-, *Armoracia*-, *Helleborus*-hybrids	>85–95% integrity at pH 1.2–7.4, 37 °C, 24–48 h	[4,5,6,9,10,18,23,59,60,113,114,115,116,117]
biocompatibility (hemolysis/cytokine ELISA)	hemolysis < 5%; IL-6, TNF-α, IFN-γ < baseline	turmeric phytosomes, ginseng PDEVs	no complement activation or macrophage stimulation at 500–1000 μg/mL	[11,12,19,20,23]
cytotoxicity (MTT/LDH/ Alamar Blue)	viability > 92% at 150 μg/mL; IC_50_ > 500 μg/mL	oregano, cinnamon PDEVs; ashwagandha PBNPs; quercetin–AuNPs; *Sideritis*–clinoptilolite hybrid; *Chelidonium*–AuNP hybrid	low lethality; superior to cationic NPs	[16,17,18,23,58,117]
genotoxicity/mutagenicity (Comet/micronucleus/Ames)	olive tail moment < 5%; no micronuclei; Ames negative	neem, moringa, silymarin–Fe_3_O_4_ hybrids	no DNA damage, chromosomal aberrations, or mutagenic effects at therapeutic doses	[19,20,23]

Data reflect physicochemical integrity (size retention, PDI), cellular and systemic compatibility (hemolysis, cytokine response), and safety outcomes (viability, IC_50_, genotoxicity) under standardized preclinical evaluation conditions. AuNPs: Gold nanoparticles; DLS: Dynamic light scattering; DNA: Deoxyribonucleic acid; ELISA: Enzyme-linked immunosorbent assay; Fe_3_O_4_: Magnetite; IC_50_: Half maximal inhibitory concentration; IFN-γ: Interferon-gamma; IL-6: Interleukin-6; LDH: Lactate dehydrogenase; MTT: 3-(4,5-Dimethylthiazol-2-yl)-2,5-diphenyltetrazolium bromide; NLCs: Nanostructured lipid carriers; NPs: Nanoparticles; NTA: Nanoparticle tracking analysis; PBNPs: Plant-based nanoparticles; PDEVs: Plant-derived extracellular vesicles; PDI: Polydispersity index; TNF-α: Tumor necrosis factor-alpha.

**Table 10:** Verified early-phase human studies involving plant-derived exosome-like components.

Plant Source/System	Clinical Identifier	Indication	Phase	Sample Size (*n*)	Primary Endpoints	Principal Findings (Preliminary/As Reported)	Status	References
grape-derived exosomes (administered as grape powder)	NCT01668849	prevention of oral mucositis in head/neck cancer chemoradiation	I/II (exploratory)	~60	mucositis incidence/severity; pain levels	suggestive reduction in mucositis symptoms in some metrics; good tolerability; no serious AEs in trial records	completed	[9,12,99,128,130,134,135,165,166,167,168,169,170,171,172,173,174,175,176,177]
plant exosomes and curcumin (generic plant-derived exosomes)	NCT01294072	curcumin delivery to normal colon tissue and colon tumors	I	~35	curcumin concentration in tissue/ tumors; safety/ tolerability	preliminary evidence of enhanced curcumin bioavailability in colon tissue; acceptable safety profile	completed (limited detailed publication)	[9,12,99,128,130,134,135,150,165,166,167,168,169,170,171,172,173,174,175,176,177]
ginger-derived exosomes (±curcumin)	NCT04879810	symptom abatement in refractory IBD	I/II (exploratory)	~20–50	safety/tolerability; symptom/disease scores; inflammation biomarkers	good tolerability; modest preliminary shifts in inflammation biomarkers and microbiota; no immunogenicity	completed (preliminary/unpublished in full detail)	[9,12,99,128,130,134,135,165,166,167,168,169,170,171,172,173,174,175,176,177]

AEs: Adverse events; IBD: Inflammatory bowel disease; *n*: Number of participants; NCT: National Clinical Trial (number).

**Table 11:** Key early-phase human trials investigating PDEVs: interventions, cohorts, and preliminary safety/tolerability outcomes.

Clinical Trial	Intervention	Status/Key Findings	References
NCT01668849	grape-derived exosomes–oral mucositis	recruitment completed; registry reports suggest modest mucositis reduction; good tolerability; full peer-reviewed publication unavailable	[183]
NCT01294072	plant exosomes for curcumin delivery to colon/tumor tissue	preliminary evidence of enhanced bioavailability; detailed peer-reviewed report limited	[178,179]
NCT04879810	ginger/curcumin exosomes in refractory IBD	completed; modest biomarker/microbiota shifts; no immunogenicity; full peer-reviewed results pending	[141,184]

IBD: Inflammatory bowel disease; NCT: National Clinical Trial (number); PDEVs: Plant-derived extracellular vesicles.

**Table 12:** Current methodologies for efficient isolation and large-scale production PDEVs: scalability, technical complexity, advantages and limitations.

Method	Description	Scale	Technical Complexity	Advantages	Limitations	References
DUC	sequential centrifugation to separate EVs from plant extracts	lab scale	medium	widely used; good for purity	time-consuming; low yield	[9,11,101]
DGUC	EVs separated based on buoyant density	lab scale	high	high purity; preserves EVs integrity	low throughput; labor-intensive	[10,13]
UF/TFF	membrane-based concentration and purification	pilot to industrial scale	medium	scalable; reproducible	membrane fouling; requires optimization	[7,197,198]
SEC	separation based on EVs size	lab to pilot scale	medium	high purity; preserves bioactivity	limited throughput	[9,11]
precipitation-based methods	polymeric reagents to aggregate EVs	lab scale	low	simple; does not require specialized equipment	co-precipitation of contaminants; less pure	[13,101]
IA capture	Ab-based EVs isolation	lab scale	high	high specificity for target EVs subpopulations	expensive; limited scalability	[7,13]
bioreactor-based production	continuous culture of plant cells or spheroids	industrial scale	high	scalable; controlled environment; defined cargo	high cost; requires optimization	[196,199]
physicochemical modulation (e.g., pH, shear stress)	adjusting culture conditions to enhance EVs yield	industrial scale	high	increased yield; maintains EVs functionality	requires precise control; cell-specific	[197,199]

Ab: Antibody; DGUC: Density gradient ultracentrifugation; DUC: Differential ultracentrifugation; EVs: Extracellular vesicles; IA: Immunoaffinity; PDEVs: Plant-derived extracellular vesicles; SEC: Size-exclusion chromatography; TFF: Tangential flow filtration; UF: Ultrafiltration.

**Table 13:** CRISPR and AI strategies for PDEVs optimization: risks, mitigation, and model construction.

Strategy	Key Objective	Potential Risks/ Challenges	Mitigation/Model Construction	References
CRISPR/Cas editing	enhance vesicle yield, tailor cargo, reduce heterogeneity	off-target effects, unintended phenotypes, epigenetic instability, ecological impacts	high-fidelity Cas variants, base/prime editors, optimized guide RNAs, vesicle-mediated CRISPR delivery, whole-genome sequencing, multi-generational phenotyping	[2,88,200,201,202,203,204,205,206]
AI-driven process optimization	improve reproducibility, yield consistency, scale-up	sparse plant-specific datasets, interpretability of black-box models, scalability validation	hybrid modeling: mechanistic (kinetics, mass balance) and data-driven (ML, DL); reinforcement learning for dynamic control; transfer learning from mammalian EVs; multi-omics and physicochemical input data	[3,4,5,88,200,201,202,203,204,205,206,207,208]

AI: Artificial intelligence; Cas: CRISPR-associated protein; CRISPR: Clustered regularly interspaced short palindromic repeats; DL: Deep learning; EVs: Extracellular vesicles; ML: Machine learning; PDEVs: Plant-derived extracellular vesicles; RNAs: Ribonucleic acids.

## Data Availability

The original contributions presented in this study are included in the article. Further inquiries can be directed to the corresponding authors.

## Abbreviations

The following abbreviations are used in this manuscript:

3D	Three-dimensional
Ab	Antibody
AEs	Adverse events
AFM	Atomic force microscopy
Ag	Silver
AgNPs	Silver nanoparticles
AI	Artificial intelligence
Au	Gold
AUC_0–*t*_	Area under the curve from time zero to the last measurable concentration (at time *t*)
AuNPs	Gold nanoparticles
AWF	Apoplast washing fluid
BBB	Blood–brain barrier
Cas	CRISPR-associated protein
C_max_	Maximum concentration
CMC	Chemistry, manufacturing, and controls
CNS	Central nervous system
CQAs	Critical quality attributes
cRGD	Cyclic arginine-glycine-aspartate
CRISPR	Clustered regularly interspaced short palindromic repeats
dcTFF	Double-cyclic tangential flow filtration
DGUC	Density gradient ultracentrifugation
DL	Deep learning
DLS	Dynamic light scattering
DNA	Deoxyribonucleic acid
DOX	Doxorubicin
DUC	Differential ultracentrifugation
EDC	1-Ethyl-3-(3-dimethylaminopropyl)carbodiimide
EGCG	Epigallocatechin-3-gallate
ELISA	Enzyme-linked immunosorbent assay
EMA	European Medicines Agency
EPR	Enhanced permeability and retention
EVs	Extracellular vesicles
FDA	Food and Drug Administration
Fe_3_O_4_	Magnetite
FTIR	Fourier-transform infrared
GC–MS	Gas chromatography–mass spectrometry
GI	Gastrointestinal
GIPCs	Glycosyl-inositol-phospho-ceramides
GLP	Good Laboratory Practice
GMO	Genetically modified organism
GMP	Good Manufacturing Practices
GO	Graphene oxide
GRAS	Generally recognized as safe
GTPases	Guanosine triphosphatases
HNTs	Halloysite nanotubes
IA	Immunoaffinity
IBD	Inflammatory bowel disease
IC_50_	Half maximal inhibitory concentration
IFN-γ	Interferon-gamma
IL-6	Interleukin-6
IP	Intellectual property
ISEV	International Society for Extracellular Vesicles
LC–MS/MS	Liquid chromatography–tandem mass spectrometry
LCA	Life cycle assessment
LDH	Lactate dehydrogenase
lncRNAs	Long non-coding ribonucleic acids
MAPK	Mitogen-activated protein kinase
miRNAs	Micro-ribonucleic acids
MISEV	Minimal Information for Studies of Extracellular Vesicles
ML	Machine learning
MRI	Magnetic resonance imaging
mRNA	Messenger ribonucleic acid
MSNPs	Mesoporous silica nanoparticles
MTT	3-(4,5-Dimethylthiazol-2-yl)-2,5-diphenyltetrazolium bromide
NAFLD	Non-alcoholic fatty liver disease
NCT	National Clinical Trial (number)
NF-κB	Nuclear factor-kappaB
NHS	*N*-hydroxysuccinimide
NLCs	Nanostructured lipid carriers
NMR	Nuclear magnetic resonance
NPs	Nanoparticles
Nrf2	Nuclear factor erythroid 2-related factor 2
NTA	Nanoparticle tracking analysis
PBNPs	Plant-based nanoparticles
PA	Phosphatidic acid
PC	Phosphatidylcholine
PCT	Patent Cooperation Treaty
PD	Pharmacodynamic
PDEVs	Plant-derived extracellular vesicles
PDI	Polydispersity index
PDT	Photodynamic therapy
PE	Phosphatidylethanolamine
PEG	Polyethylene glycol
PELNPs	Plant-derived exosome-like nanoparticles
PI	Phosphatidylinositol
PI3K/Akt	Phosphoinositide 3-kinase/protein kinase B
PK	Pharmacokinetic
PLGA	Poly(lactic-*co*-glycolic acid)
PMDA	Pharmaceuticals and Medical Devices Agency
PS	Phosphatidylserine
RECIST	Response evaluation criteria in solid tumors
RNA	Ribonucleic acid
ROS	Reactive oxygen species
RuBisCO	Ribulose-1,5-bisphosphate carboxylase/oxygenase
SANS	Small-angle neutron scattering
SAXS	Small-angle X-ray scattering
SEC	Size-exclusion chromatography
SEM	Scanning electron microscopy
siRNA	Small interfering ribonucleic acid
SLNPs	Solid lipid nanoparticles
*t*_1/2_	Half-life
TEM	Transmission electron microscopy
TET8	Tetraspanin 8
TFF	Tangential flow filtration
TIBs	Temporary immersion bioreactors
TNF-α	Tumor necrosis factor-alpha
TRPS	Tunable resistive pulse sensing
UAE	Ultrasound-assisted extraction
UF	Ultrafiltration
UV	Ultraviolet
WH	Wound healing
ZnO	Zinc oxide
